# Gene expression in metastatic breast cancer—patterns in primary tumors and metastatic tissue with prognostic potential

**DOI:** 10.3389/fmolb.2023.1343979

**Published:** 2024-02-21

**Authors:** Julia Tutzauer, Anna-Maria Larsson, Kristina Aaltonen, Caroline Bergenfelz, Pär-Ola Bendahl, Lisa Rydén

**Affiliations:** ^1^ Department of Clinical Sciences Lund, Division of Oncology, Lund University, Lund, Sweden; ^2^ Division of Translational Cancer Research, Department of Laboratory Medicine, Lund University, Lund, Sweden; ^3^ Division of Experimental Infection Medicine, Department of Translational Medicine, Lund University, Malmö, Sweden; ^4^ Department of Clinical Sciences Lund, Division of Surgery, Lund University, Lund, Sweden; ^5^ Department of Surgery, Skåne University Hospital, Malmö, Sweden

**Keywords:** metastatic breast cancer, gene expression, prognosis, primary tumors, lymph node metastasis, distant metastasis

## Abstract

**Background:** Metastatic breast cancer (MBC) is the main cause of breast cancer-related death. The outcome of MBC varies, and there is a lack of biomarkers to aid in prognostication. The primary aim of this study was to evaluate the prognostic value of gene expression (GEX) signatures in the primary tumor (PT) and distant metastasis (DM) for progression-free survival (PFS) and overall survival (OS). The secondary aim was to describe GEX changes through MBC evolution and to identify MBC subtypes.

**Methods:** RNA was extracted from the PT, lymph node metastasis (LNM), and DM from MBC patients in a prospective observational study (*n* = 142; CTC-MBC NCT01322893) and was subjected to GEX analysis retrospectively using the NanoString Breast Cancer 360™ panel. 31 continuous GEX variables in DMs and PTs were analyzed for PFS and OS by Cox regression analysis and Kaplan-Meier estimates. Multivariable Cox regressions were adjusted for number of DM sites and CTCs, visceral metastasis, ECOG status, age at MBC diagnosis and, in additional analyses, PAM50 subtype. Differential GEX analyses and Euclidean distances were used to describe subgroup differences and visualize within-patient heterogeneity.

**Results:** Compared to DM GEX, GEX of the PT was at least equally useful for predicting MBC outcome. The strongest marker for a favorable PFS, both when expressed in the PT and the DM was *AR*, even after adjustment for prognostic markers including PAM50. GEX signatures related to hormone responsiveness, including *ESR1*, *FOXA1*, *PGR*, and *AR* were favorable prognostic markers, and the p53 signature was unfavorable for PFS when expressed in PT or DM. The previously published PAM50MET signature was prognostic for both PFS and OS. We established five distinct DM GEX profiles where two associated with liver and bone metastases, respectively. Finally, we identified four DM GEX profiles able to identify MBCs with poor OS in this cohort.

**Conclusion:** GEX of both DM and PT are useful in MBC prognostication. GEX of *AR* adds prognostic information for MBC. Our descriptive analyses illuminate the biological differences between MBCs in relation to outcome and metastatic site.

## 1 Introduction

Even though advancements in diagnostics and treatments have improved survival in primary breast cancer, 20%–30% of all breast cancer patients will eventually develop a metastatic disease (Valachis et al., 2022). Metastatic breast cancer (MBC) is for most patients a disease without curative treatment options, and remains the foremost cause of breast cancer-related death. While the median overall survival of MBC is estimated to approximately 3 years (Grinda et al., 2021; Valachis et al., 2022), a considerable variability in prognosis and disease progression underscores a significant biological heterogeneity between patients. Despite this, there is a lack of prognostic biomarkers specifically tailored for MBC. The molecular biomarkers estrogen receptor alpha (ER) and human epidermal growth factor receptor 2 (HER2), initially linked to prognosis in primary breast cancer, are routinely used for prognostication and treatment prediction in MBC. The addition of gene expression (GEX) profiling, including the PAM50 subtypes, Prosigna^®^, OncoTypeDX^®^, and Mammaprint^®^, are useful in prognostication of primary breast cancer, but these profiles have not been validated for MBC. Therefore, there is a need for new biomarkers to improve prognostication and enable more personalized treatment approaches.

The marked diversity characterizing the clinical course of MBC is mirrored in its biological landscape. Another relevant contributing factor to this heterogeneity is the metastatic site (Brasó-Maristany et al., 2022), which furthermore is known to carry prognostic information. Notably, patients with bone metastasis as the exclusive metastatic site generally exhibit more favorable outcomes across all established molecular breast cancer subtypes (Bertho et al., 2021). Various models addressing the relationship between GEX and metastatic site of breast cancer have been formulated, serving both predictive purposes (Albaradei et al., 2021) and for exploratory elucidation of the biological underpinnings of the metastatic niches (Brasó-Maristany et al., 2022). However, study cohorts have overall been limited in size, methodologies have varied between studies, and findings have rarely been validated in independent cohorts. As a result, the molecular identities of MBC remain poorly elucidated and are of limited clinical use in prognostication of the individual patient.

In this study, we used a unique MBC cohort encompassing biopsies from both the primary tumor (PT), lymph node metastasis (LNM; defined as regional lymph node metastasis diagnosed and retrieved at the time of primary surgery for the PT), and distant metastasis (DM), to address the primary aim of evaluating the prognostic significance of GEX in MBC as candidates for clinical implementation.

This unique material also provides an opportunity to investigate MBC in terms of DM subtypes and tumor evolution over time. Thus, the secondary aim was exploratory: to decipher GEX in the MBC setting; to describe its successive changes through MBC evolution, from PT and LNM to the DM. Finally, using data from the secondary aim, we sought to identify biologically relevant molecular subtypes identified in MBC and investigate their prognostic potential.

The identification of novel prognostic markers and an enhanced comprehension of the molecular variants of MBC have great potential to enhance prognostic accuracy for the individual patient, to enable personalized treatment strategies, and could lead to the identification of novel targets for drug development.

## 2 Materials and methods

### 2.1 Study population

**FIGURE 1:**
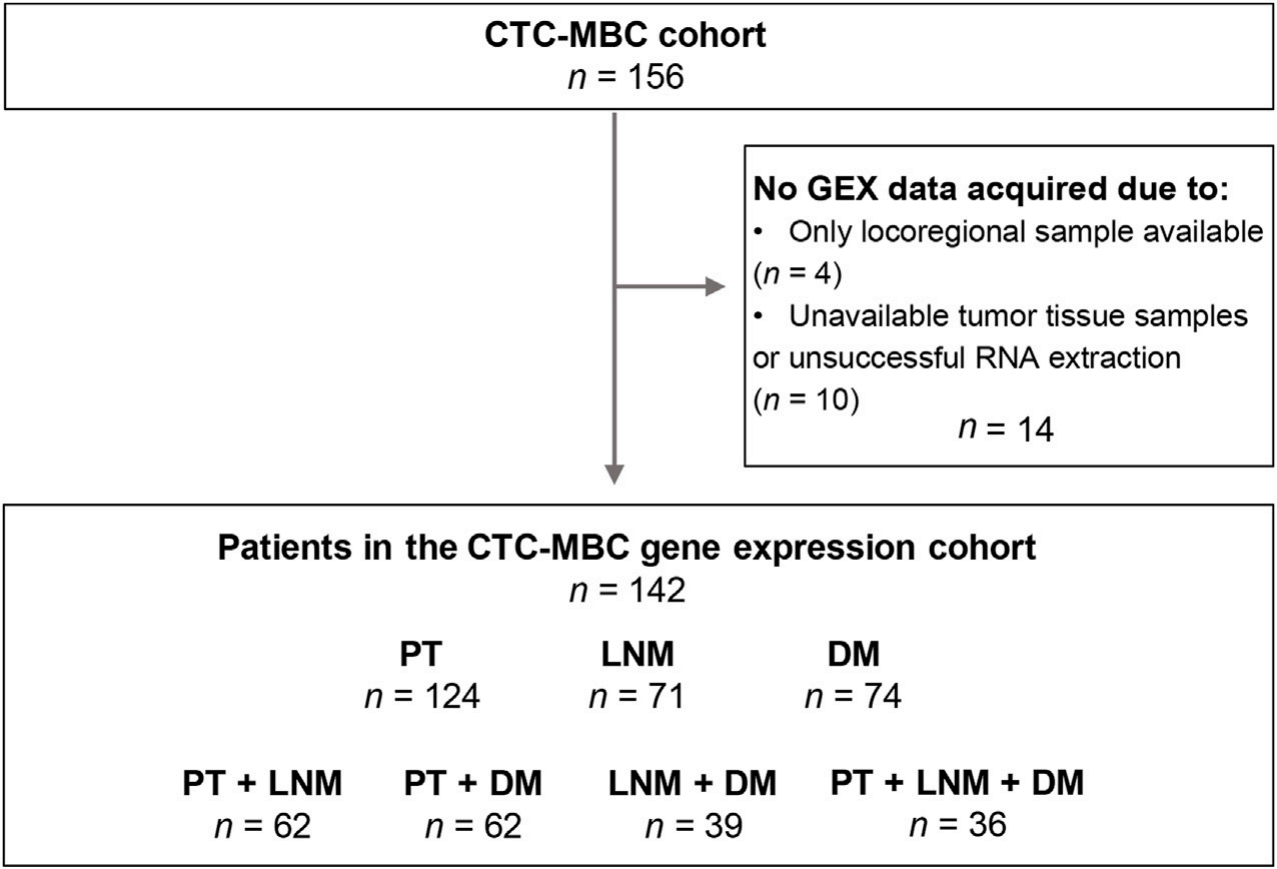
Inclusion flowchart of the study. CTC-MBC, circulating tumor cells in metastatic breast cancer; GEX, gene expression; PT, primary tumor; LNM, lymph node metastasis; DM, distant metastasis.

The current study is based on a cohort of MBC patients enrolled in the prospective observational CTC-MBC trial focusing on circulating tumor cells (CTCs), available at Clinical-Trials.gov, NCT01322893 (Larsson et al., 2018). The inclusion criteria were a diagnosis of MBC with a life expectancy of >2 months, ECOG performance status 0–2, and an age of 18 years or older. Patients were excluded if unable to understand the study information, if they had been diagnosed with other malignant disease in the past 5 years, or if they had undergone prior systemic treatment for metastatic disease. A detailed flowchart for the patients and material used in the current study is shown in Figure 1. In total, 156 patients fulfilled the inclusion criteria and were enrolled in the original study.

LNMs were defined as regional lymph node metastasis diagnosed at the time of diagnosis of the PT. Information regarding the biopsy sites of the DMs were acquired from pathology reports. Metastatic biopsies labeled “Lung” included DMs from the lung or pleura, whereas the label “Bone” included metastases from the bone or the bone marrow. Only non-regional lymph node metastasis that were obtained at the time of the diagnosis of DM were included to the group of metastatic biopsies classified to originate from “Lymph node.”

Immunohistochemical staining of ER, PR, and HER2 on tumor samples were performed according to clinical standard practice and assessed by board-certified pathologists (Larsson et al., 2018). HER2 status was considered positive if amplified by *in situ* hybridization or defined as 3+ by immunohistochemistry.

### 2.2 GEX analysis

Macrodissection of tumor tissue and RNA isolation has been described in detail previously (Jørgensen et al., 2021). Briefly, areas with representative invasive breast carcinoma tissue were selected from formalin-fixed, paraffin-embedded tumor sections and extracted by microdissection. After RNA extraction, GEX was quantified on a NanoString nCounter^®^ SPRINT Profiler (NanoString Technologies Inc., Seattle, WA, United States) using the NanoString Breast Cancer 360™ assay (BC360), which has been described in detail earlier (Jørgensen et al., 2021).

In total, GEX data were successfully acquired for *n =* 269 tumor samples: *n* = 124 PT, *n* = 71 LNM, and *n* = 74 DM samples from *n* = 142 patients (Figure 1). In cases where GEX data acquired from duplicate biopsies from the same tumor was available, the mean GEX data of the duplicates was used (*n* = 21 PT and *n* = 1 LNM). When patients had GEX data from biopsies of multiple DMs (*n* = 7), only the biopsy from the chronologically first diagnosed DM was included. Matched PT-LNM pairs were available for 62 patients, PT-DM pairs for 62 patients, LNM-DM pairs for 39 patients, and PT-LNM-DM triplets for 36 patients.

The expression of GEX signatures and single genes were normalized to *z*-scores using the sample mean and standard deviation (SD). At the time of data acquisition, the BC360 assay covered 757 individual genes (where six could not be evaluated; *CETN2, CDK4, NEIL2, PMS2, ERCC1,* and *PDCD1*) and 22 multigene signatures, including the four PAM50 signatures. The PAM50 signatures were excluded from exploratory and prognostic analyses as their prognostic value in this cohort has been described previously (Jørgensen et al., 2021). In total, 31 GEX variables were included in the prognostic analyses; 19 multigene signatures and 12 single genes, as defined by the BC360 biological signature set. Supplementary Table S1 contains additional information on the BC360 panel, including a description of the multigene signatures and their acronyms (Supplementary Table S1, Sheet 1), all the individual genes included in the panel (Supplementary Table S1, Sheet 2), and all genes constituting the GEX signatures of the biological signature set (Supplementary Table S1, Sheet 3).

### 2.3 Correlation plots

Correlation plots were created using the *corrplot* R package.

### 2.4 Gene ontology enrichment analyses

Gene Ontology (GO) enrichment analyses were performed using the R packages *clusterProfiler* and *org. Hs.eg.d*, from the biological processes subontology using a cutoff at *p =* 0.01. The functional analyses were visualized by dot plots and gene-concept networks using the R package *enrichplot*.

### 2.5 GEX clustering and heatmaps

To identify GEX patterns, we employed visualization by heatmap and k-means clustering. For more stable clustering of single genes, we focused on the 100 genes with the highest standard deviation (SD). The heatmap was generated using the R package *ComplexHeatmap* based on DM GEX data transformed to *z*-scores. The clusters in rows and columns of heatmaps as well as the cluster profiles used for prognostic evaluation were rendered using k-means clustering, based on 1000 repeated runs for optimal reproducibility.

### 2.6 Phylogenetic trees and enrichment analyses

GEX data from triplets of PT, LNM, and DM (*n* = 36) were extracted and transformed to *z*-scores. Based on all 751 genes, distance metrics between PT, LNM, and DM were calculated for each patient using Euclidean distance. Phylogenetic trees were generated by neighbor-joining (NJ) estimation using the R package *ape* and visualized using *ggtree*. To illustrate the PAM50 status of the tumors in the phylogenetic trees, we used the scores from the PAM50 analysis provided by NanoString. These scores range between 0–1, with a high score indicating that the tumor is similar to the subtype, and a low score indicate that the tumor is less similar to the subtype.

### 2.7 Differential gene expression analysis

Differential GEX analysis by linear models were performed using the R package *limma*. Genes with an FDR-adjusted *p*-value (the *q*-value) below 0.05 were considered differentially expressed. Additional methodological details are found in the Supplementary Tables S2, S3.

### 2.8 Stepwise logistic regression model predicting bone metastases

Following differential GEX analysis, differentially expressed genes were subjected to a stepwise logistic regression algorithm minimizing the models’ Akaike information criterion (AIC) by backward selection, using the R package *MASS*, allowing 10 000 steps. The binary outcome variable was whether the tumor metastasized to bone or not. Luminal status of the PT was included in the stepwise regression, but not forced into the final model. The discriminatory performance of the final model was illustrated using a ROC curve and summarized as the area under curve (AUC). To address the likelihood that this model was overfitted, we repeated the prediction model development pipeline 100 000 times with random permutations of the outcome variable. A higher AUC for the true model compared to the mean of the 100 000 mock models was interpreted as indicative of a real signal in the true model. As references, several additional models including a reduced number of descriptors were also fitted.

### 2.9 Calculation of the PAM50MET score

The PAM50MET score was calculated as described in the original article (Prat et al., 2020). The coefficients used in the calculations are found enclosed in Supplementary Table S4. Primarily, the PAM50MET score was calculated based on DM GEX. For patients where DM GEX data was not available, GEX data from PT was used (*n* = 61), as described by the authors (Prat et al., 2020). Complete data for calculation based on DM GEX was available for almost all DM samples (*n* = 72). Additional prognostic analyses were performed in the ER+, HER2-subgroup, where the calculations were made based on *n* = 56 DM, and *n* = 25 PT. Subtype was primarily determined by the ER and HER2 status of the DM, but in lack of DM data, the subtype of PT was used.

### 2.10 Statistical analysis

For testing successive changes in GEX through tumor progression, GEX of PTs, LNMs, and DMs were compared using ANOVA. The relationship between GEX signatures and the number of CTCs measured prior to treatment for MBC was evaluated using linear regression models. GEX was assessed in relation to clinical outcome using the Cox proportional hazards model where *z*-transformed GEX variables were entered as a continuous score. Cox proportional hazards models were fitted both for the full follow-up (FU) and using a time variable truncated at 2 years after diagnosis to better meet the proportional hazards assumption. The cutoff of 2 years was based on the median overall and progression-free survival of this cohort at the most recent follow-up. The endpoints were progression-free survival (PFS) and overall survival (OS). Progression was defined as progressive disease and non-progression was defined as stable disease, partial response, or complete response according to modified Response Evaluation Criteria In Solid Tumors (RECIST) 1.1 (Eisenhauer et al., 2009; Larsson et al., 2018). The multivariable Cox regressions were adjusted for the number of metastatic sites, the presence of visceral metastasis, CTC count of ≥ 5 cells per 7.5 mL blood at the time of MBC diagnosis, ECOG performance status, and age at MBC diagnosis. Output from multivariable analyses adjusting for PAM50 subtype of the DM is available as Supplementary Tables S2, S3. A *p* < 0.05 were considered statistically interesting, but given the exploratory nature of the study, *p*-values should be carefully interpreted.

## 3 Results

### 3.1 Patient and tumor characteristics

Patient and tumor characteristics of the original cohort (Larsson et al., 2018) and the subcohort subjected to GEX analyses were compared in Table 1. The characteristics of the original cohort and the GEX cohort were well balanced, indicating that the GEX cohort is representative of the full cohort (Table 1).

**TABLE 1:** Patient and tumor characteristics in the full CTC-MBC cohort and the GEX cohort.

	Original cohort *n* (%)	GEX cohort *n* (%)
**Patients in the cohorts**	156	142
**Age at PT diagnosis (years)**		
<50	42 (26.9)	39 (27.5)
50–65	41 (26.3)	36 (25.4)
≥65	73 (46.8)	67 (47.2)
**Tumor size PT**		
1	57 (38.8)	49 (36.6)
2	51 (34.7)	48 (35.8)
3	20 (13.6)	20 (14.9)
4	19 (12.9)	17 (12.7)
Missing	9	8
**Subtype PT (IHC-based)**		
Hormone receptor-positive (ER+, HER2-)	92 (73.0)	81 (72.3)
HER2 positive	17 (13.5)	16 (14.3)
Triple-negative (ER-, HER2-)	17 (13.5)	15 (13.4)
Missing	30	30
**ER status PT**		
Positive	123 (83.7)	111 (83.5)
Negative	24 (16.3)	22 (16.5)
Missing	9	9
**HER2 status PT**		
Positive	18 (14.2)	17 (15.0)
Negative	109 (85.8)	96 (85.0)
Missing	29	29
**Endocrine treatment PT**		
Yes	96 (61.5)	87 (61.3)
No	60 (38.5)	55 (38.7)
**Chemotherapy PT**		
Yes	71 (45.5)	66 (46.5)
No	85 (54.5)	76 (53.5)
**Antibody treatment PT**		
Yes	5 (3.2)	4 (2.8)
No	151 (96.8)	138 (97.2)
**Age at DM diagnosis (years)**		
<60	48 (30.8)	45 (31.7)
60–70	45 (28.8)	39 (27.5)
≥70	63 (40.4)	58 (40.8)
**ECOG status at DM diagnosis**		
0	91 (60.7)	84 (60.4)
1	37 (24.7)	35 (25.2)
2	22 (14.7)	20 (14.4)
Missing	6	3
**Subtype DM (IHC-based)**		
Hormone receptor positive, HER2- (ER+, HER2-)	80 (70.2)	75 (70.1)
HER2+	16 (14.0)	16 (15.0)
Triple negative (ER-, HER2-)	18 (15.8)	16 (15.0)
Missing	42	35
**Metastasis-free interval (MFI) (years)**		
0 years (*De novo*)	31 (19.9)	29 (20.4)
>0, ≤3 years	28 (17.9)	25 (17.6)
>3 years	97 (62.2)	88 (62.0)
**Number of metastatic sites**		
<3	109 (69.9)	97 (68.3)
≥3	47 (30.1)	45 (31.7)
**Visceral metastases**		
Yes	91 (58.3)	84 (59.2)
No	65 (41.7)	58 (40.8)
**PFS follow-up for event-free patients (median, range) (years)**	6.3 (0.64–7.4)	5.5 (0.64–7.0)
**OS follow-up for event-free patients (median, range) (years)**	7.4 (6.2–11)	8.1 (6.2–11)

Abbreviations: CI, confidence interval; DM, distant metastasis; ER, estrogen receptor; GEX, gene expression; HER2, human epidermal growth factor receptor 2; IHC, immunohistochemistry; OS, overall survival; PFS, progression-free survival; PT, primary tumor.

*Defined as a DM, diagnosed together with the PT, i.e., with MFI = 0.

No GEX signature correlated to number of CTCs measured prior to treatment for MBC (data not shown).

### 3.2 Relationships between GEX signatures in the PT, LNM, and DM

**FIGURE 2:**
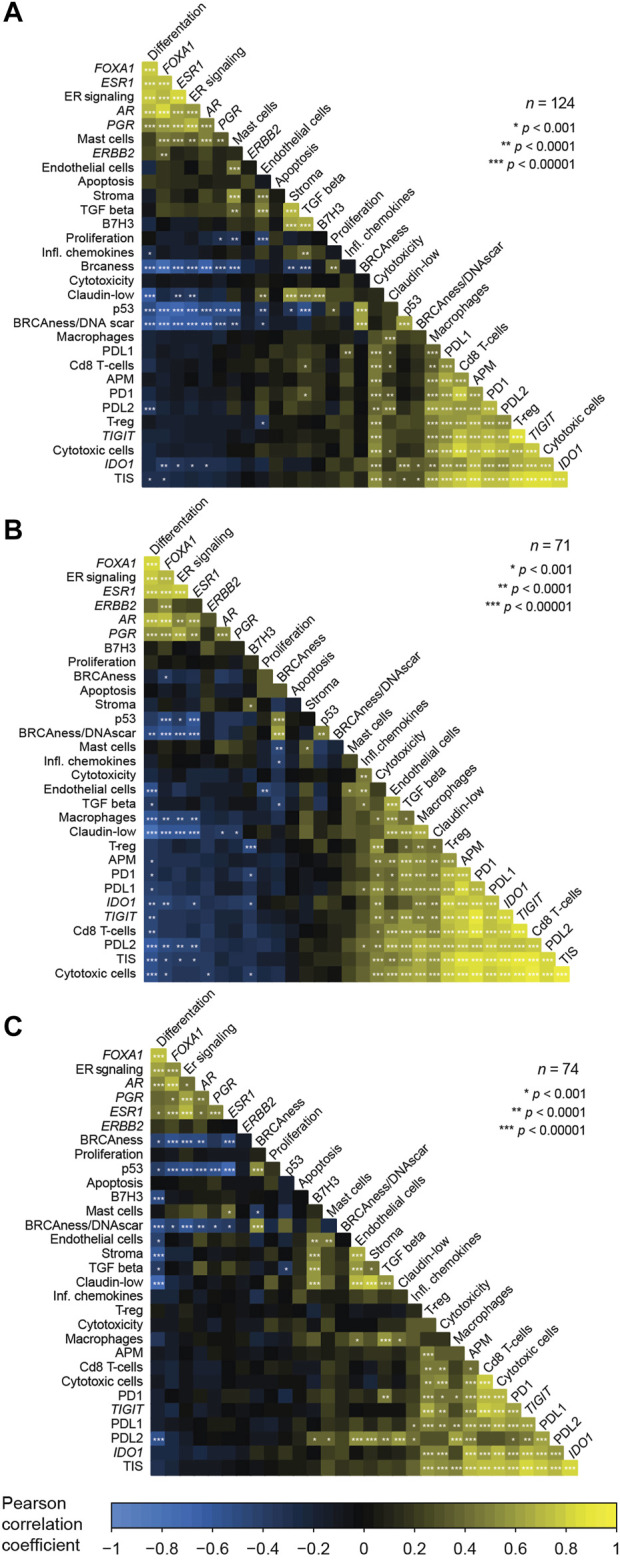
Correlations between the expression of genes and multigene signatures included in the BC360 signature set. Analyzed for each tumor site separately; the PT **(A)**, the LNM **(B)**, and the DM **(C)**. The correlation plots are ordered based on the first principal component and represent Pearson correlations between GEX of a total of 19 multigene signatures and 12 single genes standardized to z-values. Information of the abbreviations used for names of multigene signatures and which genes are included in each signature are found in Supplementary Table S1. Abbreviations: DM, distant metastasis; GEX, gene expression; LNM, lymph node metastasis; PT, primary tumor.

To improve our understanding of the interplay between the biological signatures in the BC360 panel in the context of MBC, and consequently establish a foundation that could aid in interpreting the findings of this study, we wanted to assess the relationship between the GEX signatures of the BC360 panel. To this end, we constructed correlation plots showing the GEX of genes and multigene signatures included in the biological signature set of BC360 for all three tumor sites (Figure 2). In general, the patterns were similar at all three sites. The GEX signature representing differentiation correlated strongly with a cluster of hormone-related GEX variables, including the genes *FOXA1*, *ESR1*, *AR*, and *PGR*, and the multigene signature ER signaling. In the PT, these variables all correlated strongly with the mast cell GEX signature, a trend that was not as pronounced in the LNM or DM. A second cluster represented immune-related genes, including the genes *CD274* (from here on referred to as PDL1), *PDCD1* (from here on referred to as PD1), *PDCD1LG2* (from here on referred to as PDL2), *TIGIT*, *IDO1*, and the signatures representing APM, T-reg, cytotoxic cells, macrophages, and CD8 T-cell signatures. Interestingly, the hormone-dependent GEX cluster and the immune-related gene cluster correlated negatively at all three tumor sites. The hormone-dependent genes also correlated negatively to GEX signatures related to the TNBC subtype and genetic instability, including BRCAness, claudin low, p53, and BRCAness/DNA scar, which is the BRCAness signature combined with a homologous recombination deficiency (HRD) signature. In general, the relationships between the GEX signatures followed a similar pattern at the different tumor sites. Due to the high level of correlation between the GEX signatures explored in this study, results must be carefully interpreted. More information on the multigene signatures and abbreviations used in multigene signature names is found in Supplementary Table S1.

### 3.3 Diverse GEX patterns through tumor progression

To explore the biological profile at each step of tumor progression in MBC, we compared the GEX at each tumor site including all available data from the PTs, LNMs, and DMs. First, to address broader patterns at the different sites, we plotted the biological signatures of the BC360 panel (Supplementary Figures S1A, B). The patterns for GEX of the genes and multigene signatures were in general either that GEX; 1) successively decreased through tumor progression, which was observed mainly in signatures associated to a less aggressive tumor type, including mast cells, ER signaling, and *PGR*, or 2) increase in GEX in LNM compared to PT, often to decrease below the PT level in DM, the pattern of most immune-related signatures, including APM, cytotoxic cells, *IDO1*, PD1, PDL1/2, *TIGIT*, TIS, and T-reg, as well as the apoptosis signature. A similar pattern, but where the LNM GEX instead were lower than both PT and DM, was observed for the B7H3 gene. The pattern of downregulation of *ESR1* GEX was unclear in the full cohort (Supplementary Figure S1B). In line with previous reports (Encarnación et al., 1993; Johnston et al., 1995), the *ESR1* GEX decreased through tumor progression in patients with luminal PT that had undergone adjuvant endocrine treatment for their primary breast cancer (Supplementary Figures S2, *n* = 65). However, this decrease was noticed already in the LNM, where the tumor cells are still treatment naïve.

**FIGURE 3:**
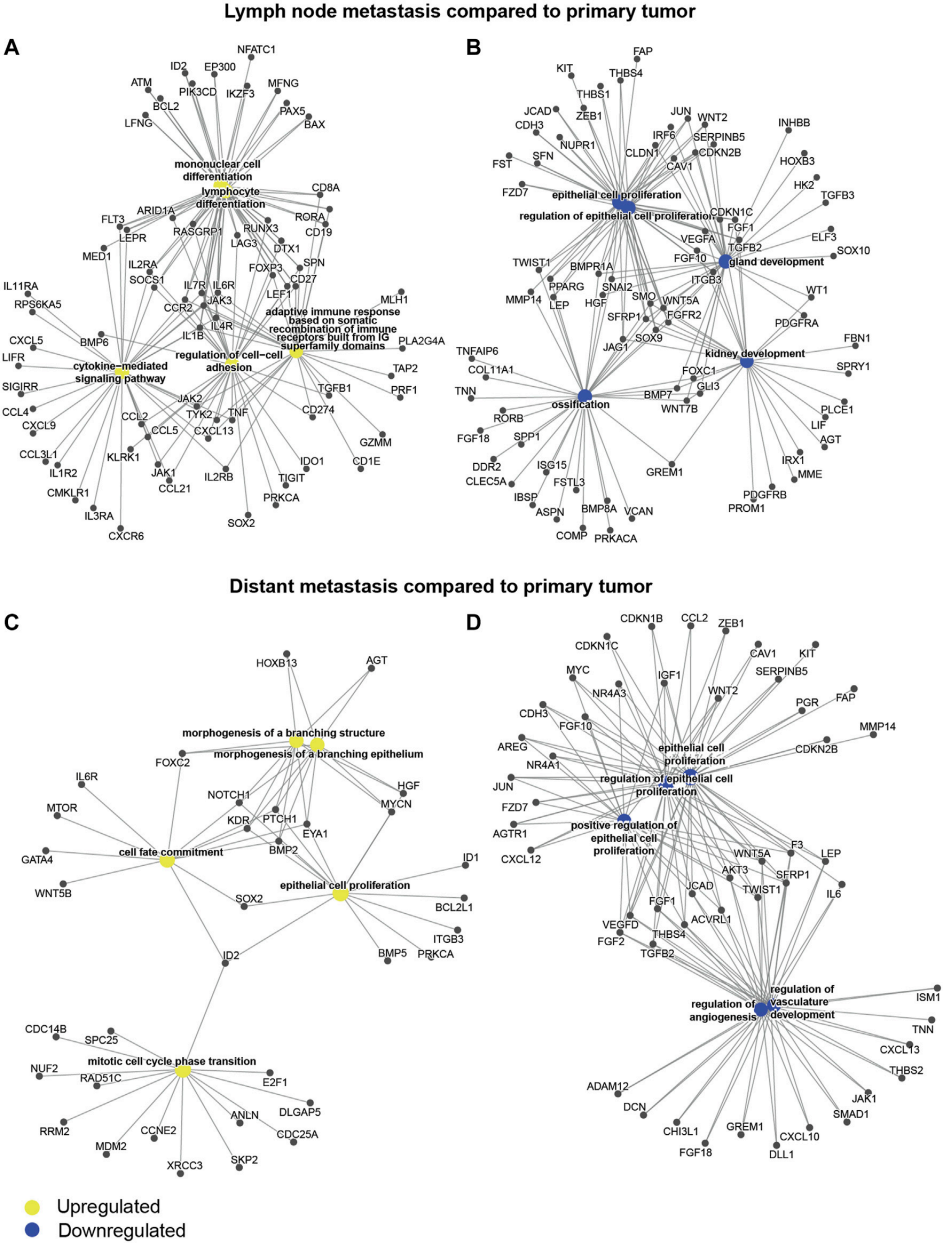
Differential gene expression through tumor evolution. Gene-concept networks presenting the results from differential gene expression analysis between tumor sites: **(A)** genes upregulated in the LNM compared to the PT, **(B)** genes downregulated in the LNM compared to the PT, **(C)** genes upregulated in the DM compared to the PT, **(D)** genes downregulated in the DM compared to the PT. The networks were based on all genes with a *q* < 0.05 from *limma* models, where the log fold change was >0 for upregulated genes **(A, C)**, or <0 for downregulated genes **(B, D)**. Abbreviations: DM, distant metastasis; LNM, lymph node metastasis; PT, primary tumor.

To address differences in GEX at the different sites at the gene level, we performed differential GEX analysis including all 751 individual genes of the BC360 panel. Genes with a *q* < 0.05 were considered differentially expressed. A total of 252 genes were differentially expressed in the DM compared to the PT, 341 genes in the DM compared to the LNM, and 333 in the LNM compared to the PT. The up- and downregulated genes in the LNM and DM compared to the PT are shown as gene-concept networks in Figure 3. Between the PT and the LNM, the functions of upregulated genes were mainly lymphocyte and mononuclear cell differentiation, cytokine signaling, adaptive immune response, and cell-cell adhesion (Figure 3A), possibly reflecting an increased immune infiltration at this site. In line with this, the most highly differentially expressed gene between PT and LNM was the frequently used B cell marker *CD19* (Wang et al., 2012), for which the expression was 8 times higher in the LNM than in the PT. The functions of downregulated genes in LNM compared to PT included, e.g. epithelial cell proliferation and gland development (Figure 3B). With regards to differences between the PT and the DM, genes upregulated in the DM compared to the PT were associated with functions such as epithelial cell proliferation, cell cycle progression, cell fate commitment, and morphogenesis of a branching epithelium (Figure 3C). Interestingly, some genes that were downregulated in the DM compared to the PT were also active in epithelial cell proliferation. Another biological function suggested to be downregulated in the DM compared to the PT was angiogenesis (Figure 3D). The most prominent GEX fold change in DM compared to PT was seen for *SFRP2*, which is involved in angiogenesis (van Loon et al., 2021), where DM expression was found to be around 6% of that observed in PTs. All genes that are differentially expressed between the sites with a *q* < 0.05 are found in Supplementary Table S2.

To summarize the GEX changes through tumor evolution of MBC, the patterns of the BC360 biological signatures illustrate how traits related to differentiation and less aggressive tumor behavior are successively downregulated through MBC progression. Both in terms of BC360 biological signatures and on the single gene level, it is clear that the tumor microenvironment (TME) of the LNM harbors an increased level of immune-related events compared to the PT. Furthermore, on the single gene level, a pattern appears that several angiogenesis-associated genes are downregulated in the DM compared to the PT.

### 3.4 Phylogenetic relationships between triplets of PT, LNM, and DM

**FIGURE 4:**
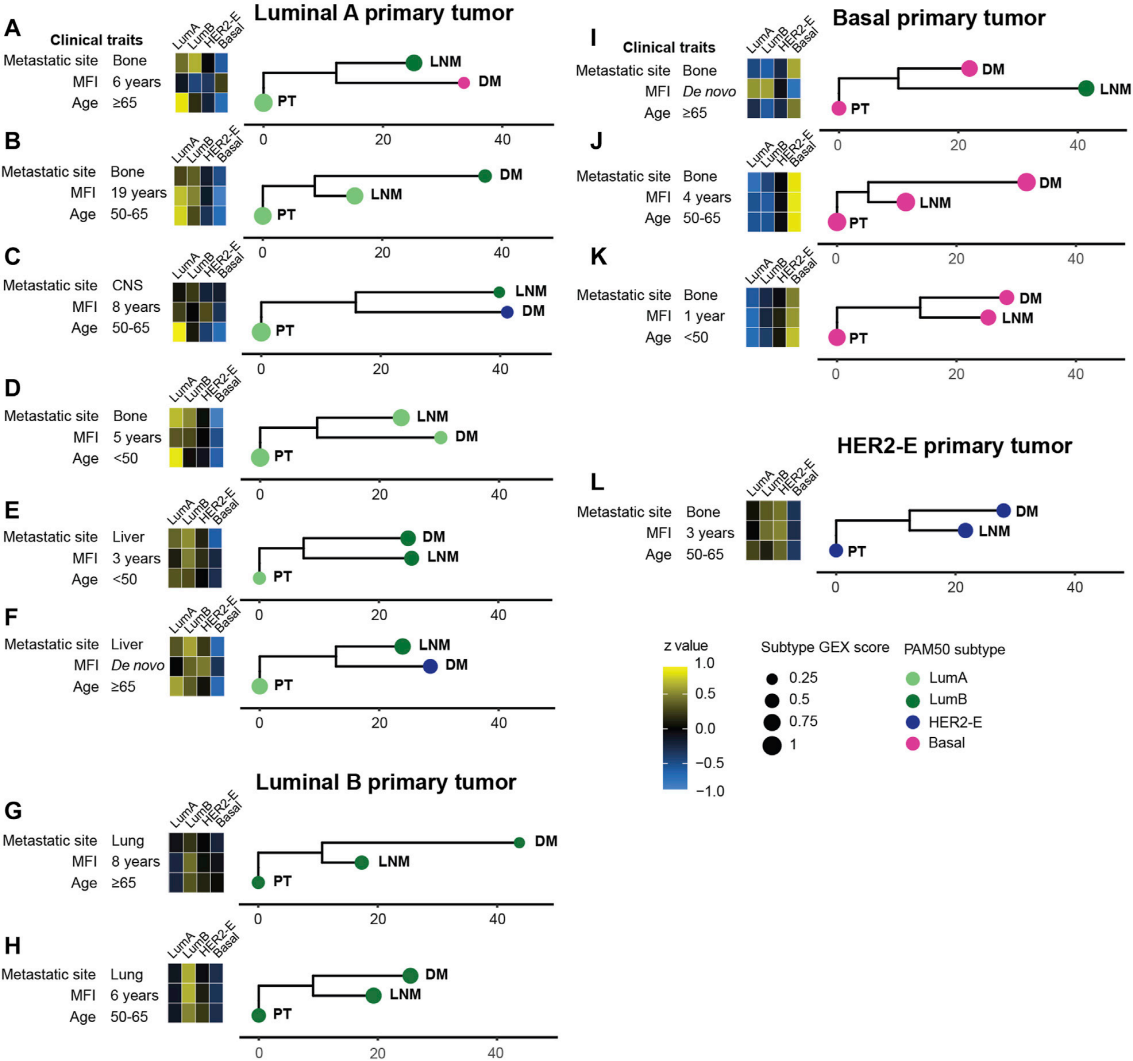
Phylogenetic trees for a selected subset of MBC triplets **(A–L)**. The relationship between tumors within patients was determined using the neighbor joining (NJ) algorithm from Euclidian distances of all 751 genes in the BC360 gene set, and manually rooted in the primary tumor. The horizontal scale bars represent the calculated Euclidian distance. Heatmaps show the gene expression of the PAM50 subtypes. The score of the PAM50 subtype, i.e., how well the GEX profile of the tumor agrees with the determined PAM50 subtype, is shown as the size of the bullet. Phylogenetic trees for the remaining MBC triplets are found in Supplementary Figures S3, S4. Abbreviations: DM, distant metastasis; LNM, lymph node metastasis; Lum, Luminal; MFI, metastasis-free interval; NJ, neighbor joining; PT, primary tumor.

To further explore the dynamic changes in GEX throughout tumor progression in different MBCs where GEX data was available for PT, LNM, and DM (*n* = 36), we calculated the GEX relatedness as Euclidean distance measures, and visualized the patterns as phylogenetic trees (Figure 4; Supplementary Figures S3, S4). Figure 4 presents representative MBC cases selected to cover a range of clinical and biological conditions. The PAM50 subtypes are presented both with the label and the subtype score calculated in the PAM50 analysis, the latter to illustrate how similar the tumor´s GEX is to that of the subtype. As previously reported for this cohort (Jørgensen et al., 2021), changes in PAM50 subtype were common through tumor progression, with a higher tendency of shifts toward a more aggressive subtype. These shifts were observed both in cases where the PT displayed a low subtype score, indicating a relatively lower similarity to the subtype, (e.g., Figures 4E,L), and cases where the PT had a high subtype GEX score, indicating a higher similarity to the subtype, (e.g., Figures 4A,C). In most patients, the PT displayed a closer relationship to the LNM than to the DM. An interesting pattern was seen in one of the *de novo* MBCs (Figure 4I), where the PT and DM were basal-like, but the LNM had a luminal B subtype and showed considerably lower GEX resemblance to the PT than the DM. Furthermore, both *de novo* MBCs with GEX data from both PT, LNM, and DM exhibited subtype shifts (Figures 4F,I). It is interesting to note that the Euclidean distance between PT and LNM of one of the *de novo* MBC was longer than that of a PT and DM diagnosed 19 years apart (Figures 4B,I). Figures 4G,H illustrates that similar metastatic site, MFI, and PAM50 subtype of all three tumors, two MBCs can exhibit different patterns in terms of GEX evolution from the time of the PT and LNM to the DM. No statistically convincing relationship was found between Euclidean GEX distance of the MBC and clinical factors such as MFI, PAM50 subtype, or age at diagnosis (data not shown). In summary, these data illustrate the versatility of MBC in terms of evolutionary relationships between tumors, that MBC often shifts to more aggressive subtypes, and suggest that the PT and LNM most often share a higher level of similarity than the PT and DM.

### 3.5 Site-predictive potential of PT gene expression for bone metastasis

Considering that the metastatic site carries prognostic information in MBC, it is of clinical interest to predict which PTs are at risk of recurring at specific sites. Therefore, to identify potential PT GEX profiles related to the site of metastatic spread, we performed differential GEX analyses for each of the more prevalent metastatic sites, stratifying on a binary variable of whether the PT spread to a particular site or not. The sites included in the analyses were skin, liver, lung, lymph nodes, and bone. After adjusting for FDR, the only metastatic site where any differential GEX had a *q* < 0.05 was bone, where 85 genes were differentially expressed in PTs that later recurred in bone at initial MBC diagnosis compared to PTs that did not recur in bone (Supplementary Table S3).

To further evaluate the site-predictive potential of the 85 genes associated to bone metastasis, we included these genes in a stepwise logistic regression in relation to the binary outcome of whether the PT metastasized to bone. As the majority of the PTs in this cohort are luminal breast cancers, which are prone to spread to bone (Kennecke et al., 2010), we adjusted the model for luminal status of the PT to prevent the risk of the final model only reflecting luminal vs. non-luminal genes. The final model is available in Supplementary Table S5. Interestingly, luminal subtype was not included in the final model, suggesting that the genes had a stronger association to bone metastasis than a luminal subtype does. GO enrichment analysis of biological function showed that the genes included in the model were mainly involved in cell cycle regulation and mitosis (Supplementary Table S5). After comparing the resulting model with several other models restricted to between four and thirty predictors, as well as 100 000 mock models developed after random permutations of the outcome variables, i.e. under the null hypothesis of no association between expression of the selected genes and outcome (Supplementary Table S5), we concluded that the final large model is most likely overfitted, but that a high model complexity is required to accurately predict bone metastases in this material. In the light of this observation and the fact that the PT population in our dataset is relatively small and does not represent a clinical setting for patients with newly diagnosed primary breast cancer, it is appropriate to interpret the data from this model as hypothesis-generating, rather than truly predictive of bone metastases.

### 3.6 GEX of DMs at different metastatic sites

**FIGURE 5:**
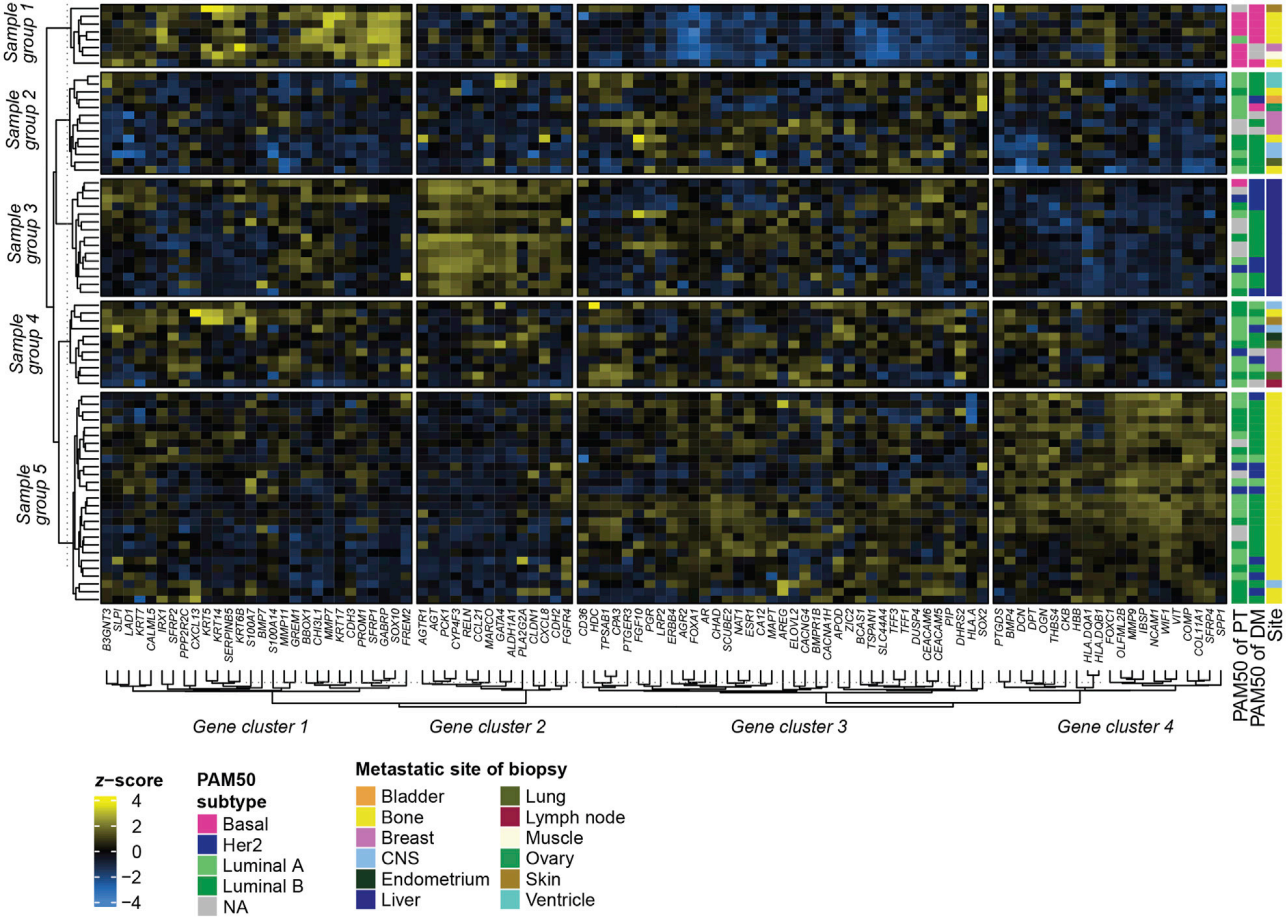
Heatmap of GEX in metastatic biopsies. Heatmap based on the 100 genes with the highest variability (SD) among the DMs. Rows represent different samples, and columns represent genes. Metastatic site refers to the site of the metastatic biopsy, not excluding that the patient may have had multiple metastatic sites. Clusters of rows (samples) and columns (genes) were rendered using k-means clustering and represent the consensus of 1000 repeated runs for optimal reproducibility. Abbreviations: DM, distant metastasis; GEX, gene expression; Lum, luminal; SD, standard deviation.

**FIGURE 6:**
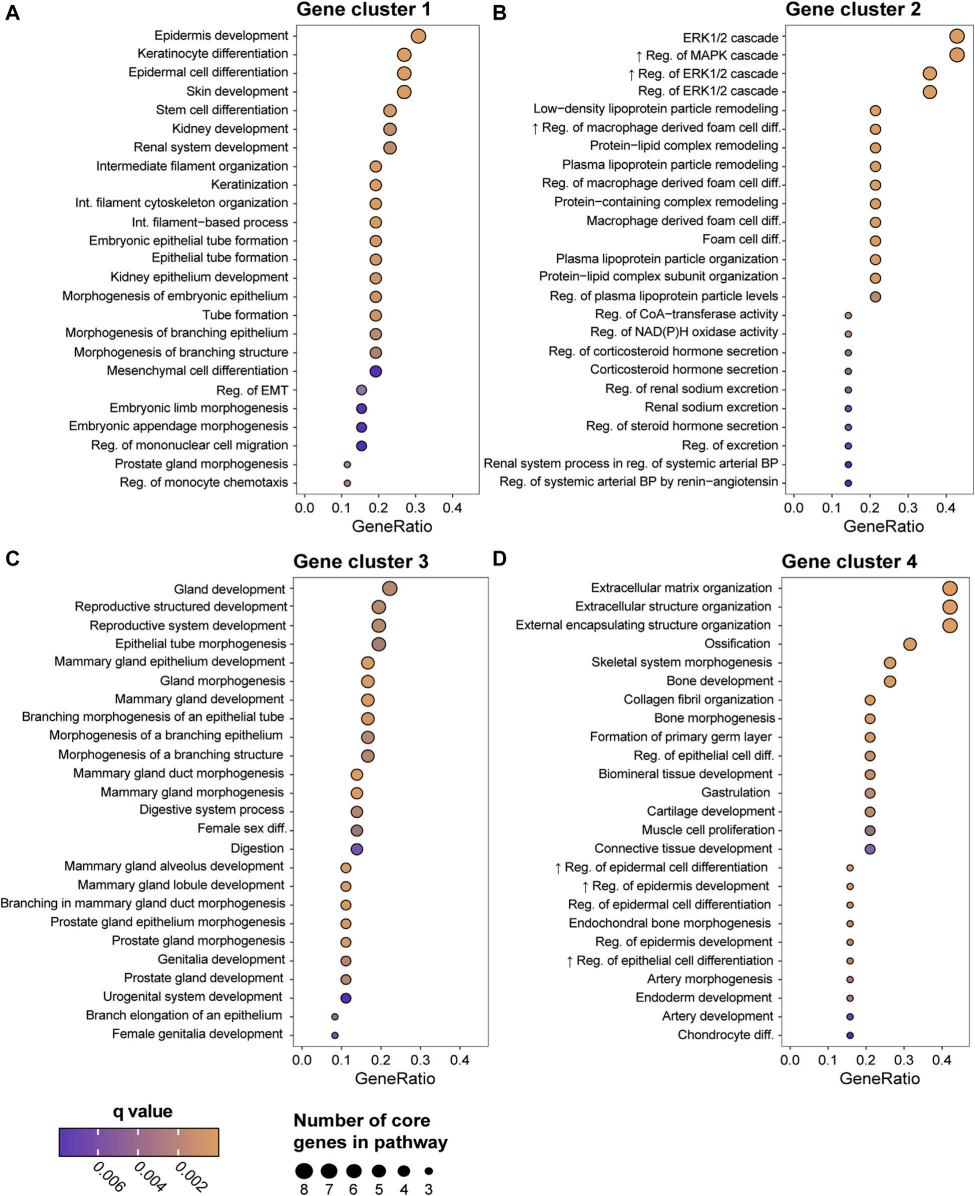
Gene Ontology (GO) enrichment analysis of the biological function of the four gene clusters generated in the Figure 5 heatmap; group 1 **(A)**, group 2 **(B)**, group 3 **(C)**, and group 4 **(D)**. Showing the top 25 biological functions based on GeneRatio, which is the ratio between the core enrichment genes and the total number of genes in the pathway and illustrates to which level the gene cluster is altered in the pathway. The number of core enrichment genes is indicated by the dot size. The *q* values represent *p* values adjusted for FDR. Abbreviations: FDR, false discovery rate; GO, Gene Ontology.

To address if the GEX of DMs is related to the metastatic site, we constructed a heatmap representing the GEX of the 100 genes of highest expression variability in the DMs (Figure 5). Four stable gene clusters were identified (Figure 5, column clusters). Gene set enrichment analysis showed that the core enrichment genes, i.e., the genes that account for the enrichment signal (Subra et al., 2005) of gene cluster 1 were related to epidermal development, embryonic processes, epithelial to mesenchymal transition (EMT), and extracellular matrix (Figure 6A). Gene cluster 2 had several genes involved in MAPK signaling, macrophage-derived foam cell differentiation, and metabolic processes involved in, e.g., lipid metabolism (Figure 6B). Cluster 3 was the largest group and most notably involved in female sex differentiation and the development of the reproductive system and mammary glands. This cluster included several genes from the hormone sensitive cluster found in the correlation plots (Figure 2), *ESR1, PGR,* and *FOXA1* (Figure 6C). Finally, gene cluster 4 was involved in processes concerning skeletal bone, including ossification, skeletal system morphogenesis, cartilage development, and chondrocyte differentiation, but also processes in the extracellular matrix (Figure 6D).

In terms of sample (row) groups, it is highly interesting to note that regardless of the PAM50 profile of the PT, all DMs residing in the liver ended up in the same group (Figure 5, Sample group 3). This group had high expression of gene cluster 2, which consists of several metabolism-regulating genes such as *PCK1*, which is considered the master regulator of gluconeogenesis, and *AGTR1* and *AGT*, which are involved in NAD(P)H oxidase activity. Similarly, sample group 5 identified a large portion of the DMs located in bone, while only including one DM from a different site. In contrast, sample group 2 and 4 included metastases from a variety of sites. Two sample groups (Albaradei et al., 2021; Valachis et al., 2022) consisting of mainly bone metastases formed in separate parts of the heatmap. Sample group 1 comprises mostly basal DMs and is highly active in gene cluster 1 and inactive in gene cluster 3, whereas sample group 5 has a high expression in gene cluster 4. It is also interesting to note that the only two ventricle DMs, both of luminal B subtype originating from a luminal A PT, clustered as nearest neighbors in sample group 2, indicating related GEX profiles. In summary, these results show that among the most variably expressed genes in our gene set, some are highly associated with the metastatic site, and to some degree with the PAM50 subtype.

### 3.7 Prognostic role of GEX in metastatic breast cancer DM

**FIGURE 7:**
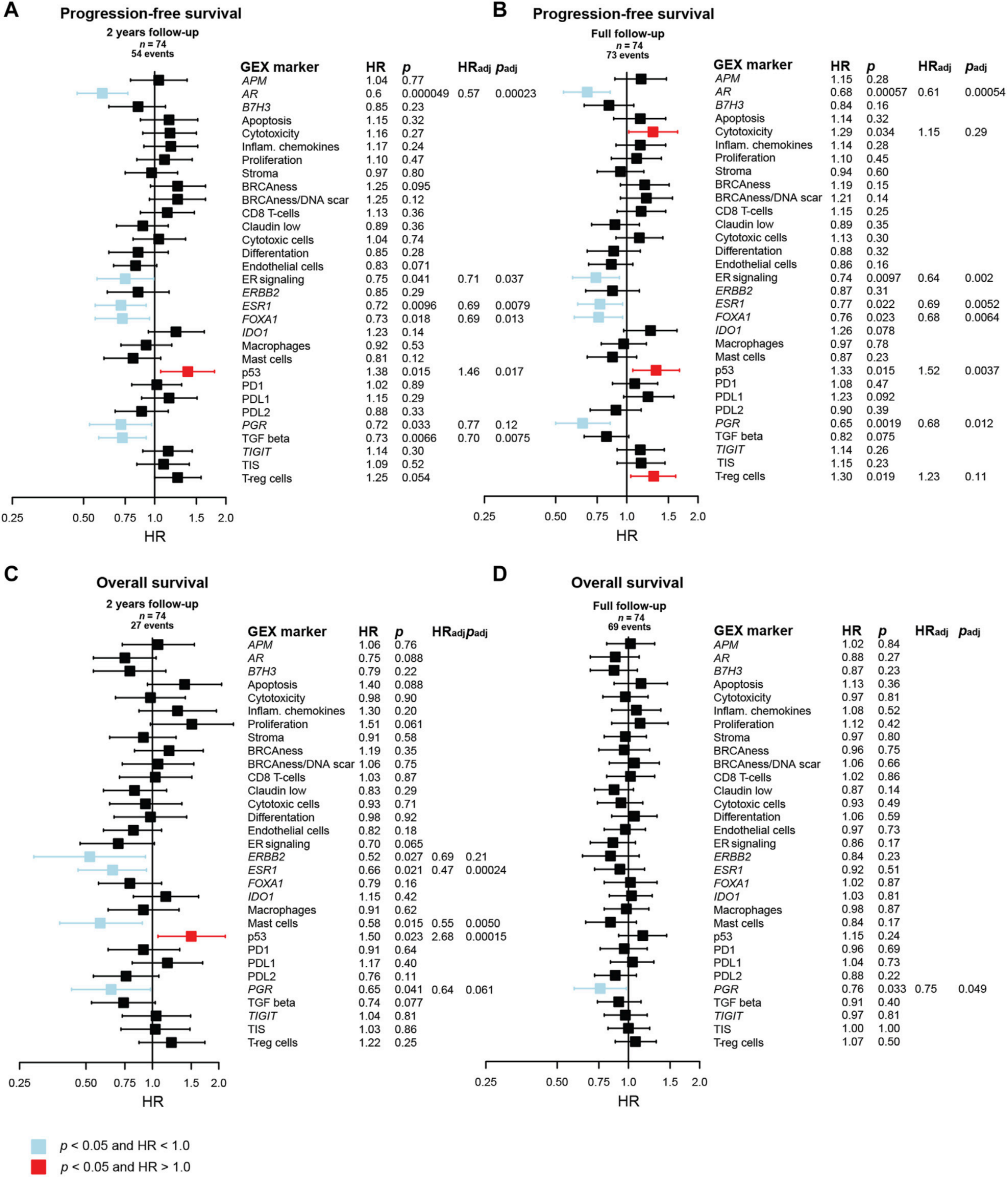
Prognostic value of the BC360 biological GEX signatures when expressed in the DM. Forest plots presenting the relationship between DM expression of the signatures and **(A)** PFS after 2 years of follow-up, **(B)** PFS after full follow-up, **(C)** OS after 2 years of follow-up, **(D)** OS after full follow-up. The plots, as well as the number of patients (*n*) and events are based on univariable Cox regression models with z-transformed GEX entered as continuous variables. HR is plotted with 95% CI. Colored based on the *p*-value. Presented *p-*values are crude. Multivariable Cox regression models were calculated for signatures with a *p* < 0.05 in the univariable Cox regression model. Adjustments were made for number of metastatic sites, visceral metastasis, ECOG performance status, number of CTC (≥5 per 7.5 mL blood at baseline) at MBC diagnosis, and age at MBC diagnosis, and the results are presented as HR_adj_ and *p*_adj_. Abbreviations: CI, confidence interval; CTC, circulating tumor cells; DM, distant metastasis; GEX, gene expression; PFS, progression-free survival; MBC, metastatic breast cancer; OS, overall survival.

The results of univariable and multivariable Cox regression models of the prognostic value of continuous DM GEX of the biological signatures in the BC360 panel are found in Figure 7. In line with previous reports for primary breast cancer (Lundgren et al., 2023), a cluster of genes related to hormone-responsiveness were associated with a decreased risk in terms of PFS, both within the first 2 years after MBC diagnosis, and when considering the full follow-up (Figures 7A,B). These genes included *ESR1* (HR_Full FU_ = 0.77, 95% CI = 0.62–0.96, *p* = 0.022)*, PGR* (HR_Full FU_ = 0.65, 95% CI = 0.50–0.85, *p* = 0.0019)*,* as well as *FOXA1* (HR_Full FU_ = 0.76, 95% CI = 0.60–0.96, *p* = 0.023) and *AR* (HR_Full FU_ = 0.68, 95% CI = 0.54–0.85, *p* = 0.00057), as well as the signature representing ER signaling (HR_Full FU_ = 0.74, 95% CI = 0.59–0.93, *p* = 0.0097). The p53 signature was found to associate with an increased risk of early progression within both 2 years and during the full follow-up (HR_Full FU_ = 1.33, 95% CI = 1.06–1.67, *p* = 0.015). Interestingly, two other GEX signatures that associated to a worse PFS when considering the full follow-up were cytotoxicity (HR_Full FU_ = 1.29, 95% CI = 1.02–1.64, *p* = 0.034) and T-reg cells (HR_Full FU_ = 1.30, 95% CI = 1.04–1.61, *p* = 0.019).

In a multivariable Cox model adjusting for number of metastatic sites, visceral metastasis, ECOG performance status, number of CTCs (≥5 per 7.5 mL blood at baseline), and age at MBC diagnosis, *ESR1*, *AR*, *FOXA1*, ER signaling, and p53 remained prognostic for PFS with *p* < 0.05 both for 2 years and full follow-up. TGF beta was prognostic for 2 years PFS after adjustment, and *PGR* was prognostic for PFS at full follow-up after adjustment (Figures 7A,B). Many genes in the BC360 GEX panel are included in the PAM50 subclassification, why no multivariable adjustment was made for PAM50 subtype in the initial Cox regression model. However, to identify factors that may add prognostic information beyond PAM50, we also fitted the multivariable Cox model including PAM50 subtype of the DM in addition to adjusting for the clinical variables mentioned above. In this analysis, *AR* and TGF beta were identified as independent prognostic factors for favorable PFS at 2 years of follow-up, and only *AR* for the full follow-up (Supplementary Figure S5).

In relation to OS after 2 years of follow-up (Figure 7C), a better outcome was observed for patients with high DM expression of *ERBB2* (HR_2yrs_ = 0.52, 95% CI = 0.29–0.93, *p* = 0.027), *ESR1* (HR_2yrs_ = 0.66, 95% CI = 0.46–0.94, *p* = 0.021), mast cells (HR_2yrs_ = 0.58, 95% CI = 0.38–0.90, *p* = 0.015), p53 (HR_2yrs_ = 1.50, 95% CI = 1.06–2.14, *p* = 0.023), and *PGR* (HR_2yrs_ = 0.65, 95% CI = 0.43–0.98, *p* = 0.041). In multivariable analysis, *ESR1*, mast cells, and p53 remained prognostic for 2 years OS. The only prognostic factor found for OS with full follow-up was *PGR* (HR_Full_ = 0.76, 95% CI = 0.59–0.98, *p* = 0.033), which was also prognostic in multivariable analysis (Figure 7D). When including PAM50 in the multivariable Cox model, only the p53 signature remained prognostic of OS, and only after 2 years of follow-up (Supplementary Figure S5).

To further explore the prognostic value of *AR* GEX of the DM, *AR* quartiles were plotted in relation to PFS and OS in Kaplan-Meier curves (Supplementary Figure S6). In line with the results from the Cox regressions of the linear *AR* variable (Figure 7), the *AR* quartiles were ordered with the highest quartile (Q4) being visually associated with the best PFS. The pattern was particularly clear during the first 2 years after diagnosis for PFS (HR_2 yrs_ = 0.44, 95% CI = 0.22–0.91, *p* = 0.027), but not for OS (HR_2 yrs_ = 0.77, 95% CI = 0.27–1.94, *p* = 0.52).

### 3.8 Prognostic role of GEX in the PT of metastatic breast cancer

**FIGURE 8:**
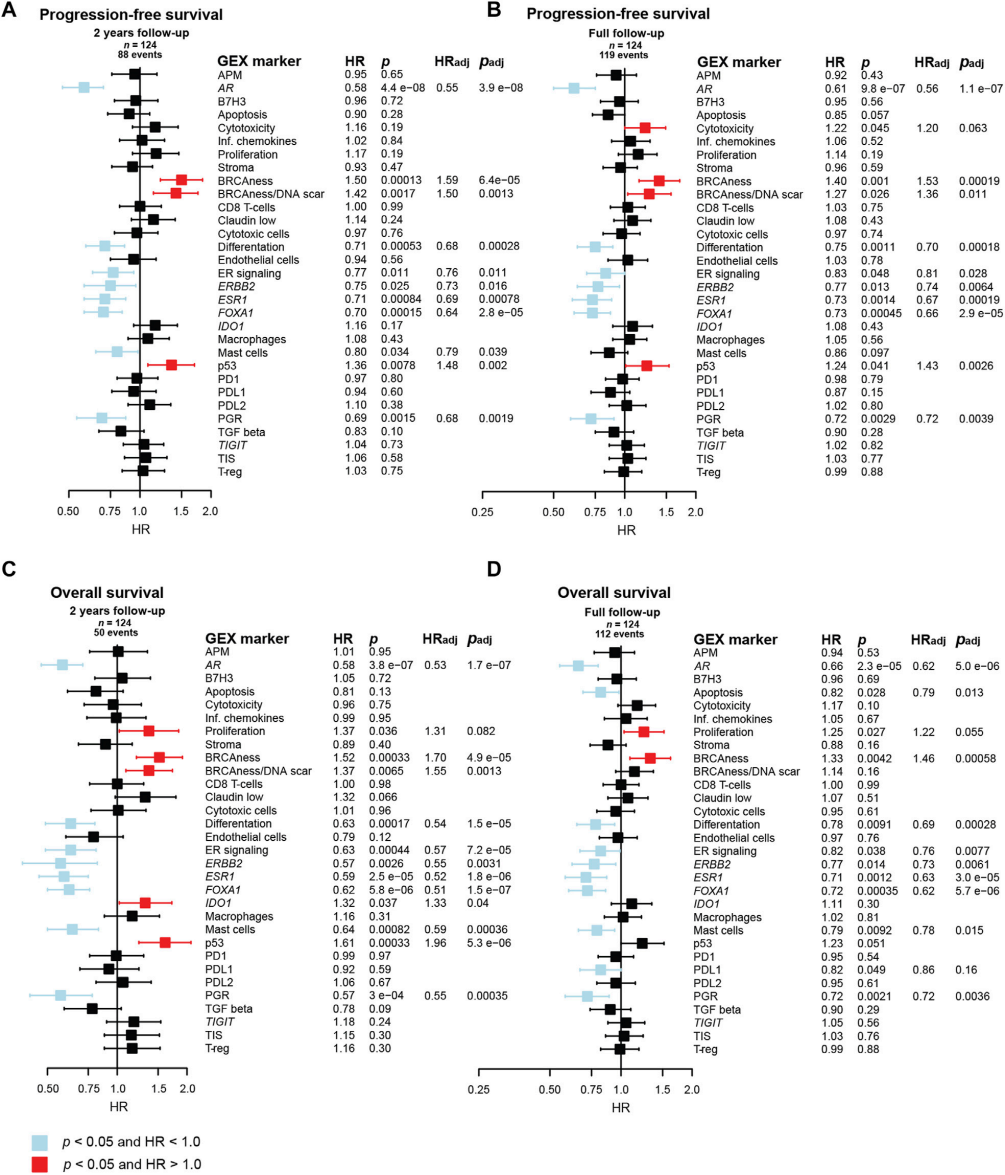
Prognostic value of the BC360 biological GEX signatures when expressed in the PT. Forest plots presenting the relationship between PT expression of the signatures and **(A)** PFS after 2 years of follow-up, **(B)** PFS after full follow-up, **(C)** OS after 2 years of follow-up, **(D)** OS after full follow-up. The plots, as well as the number of patients (*n*) and events are based on univariable Cox regression models with z-transformed GEX entered as continuous variables. HR is plotted with 95% CI. Colored based on the *p*-value. Presented *p-*values are crude. Multivariable Cox regression models were calculated for signatures with a *p* < 0.05 in the univariable Cox regression model. Adjustments were made for number of metastatic sites, visceral metastasis, ECOG performance status, number of CTC (≥5 per 7.5 mL blood at baseline) at MBC diagnosis, and age at MBC diagnosis, and the results are presented as HR_adj_ and *p*_adj_. Abbreviations: CI, confidence interval; CTC, circulating tumor cells; GEX, gene expression; PFS, progression-free survival; PT, primary tumor; MBC, metastatic breast cancer; OS, overall survival.

As tumor tissue from the DM is not always available, we also wanted to determine the prognostic value of GEX of the BC360 panel in PT in MBC (Figure 8). To address this, univariable and multivariable Cox regression models were fitted with the GEX of the biological signatures in the BC360 panel as continuous variables in relation to PFS and OS. Similar patterns to what was observed for DM GEX emerged, but interestingly, the prognostic value was even more pronounced in relation to the PT GEX.

The favorable prognostic value of the cluster of genes and GEX signatures related to hormone-responsiveness, including *AR*, ER signaling, *ESR1*, *FOXA1*, and *PGR*, was strong in relation to both PFS and OS after both 2 years and full follow-up. Similarly, the BRCAness signature emerged as a prognostically unfavorable marker for poor PFS and OS after both follow-up intervals. Similar results were observed for p53, but the effect was not as pronounced for OS after full follow-up. *ERBB2* and differentiation also associated to better PFS and OS after both follow-up intervals. Similar results were seen for the mast cell signature.

In a multivariable Cox model adjusting for number of metastatic sites, visceral metastasis, ECOG performance status, number of CTCs (≥5 per 7.5 mL blood) at baseline, and age at MBC diagnosis, all the genes and GEX signatures mentioned above remained associated to outcome with *p* < 0.05. Importantly, the strongest statistical evidence of prognostic value of PT GEX was observed for *AR*, which had *p*-values between 3.9 × 10^−8^ and 2.3 × 10^−5^ in both univariable and multivariable for both endpoints and both follow-up intervals.

As many genes in the BC360 GEX panel are included in the PAM50 subclassification, no multivariable adjustment was made for PAM50 subtype in the initial Cox regression model. However, to address if the prognostic value of the genes found to be associated to outcome was independent on PAM50 status, additional multivariable Cox regression analyses were performed. In these models, *AR* was the only gene or GEX signature that remained prognostic for 2-year PFS. Both *AR* and *ESR1* were prognostic for favorable PFS at full follow-up. Genes and GEX signatures associated to 2-year OS after adding PT PAM50 status to the Cox model were *ESR1* and p53, and after full follow-up, apoptosis, proliferation, *ESR1*, mast cells, and PDL1 remained associated to outcome (Supplementary Figure S7).

### 3.9 Prognostic performance of PAM50MET

**FIGURE 9:**
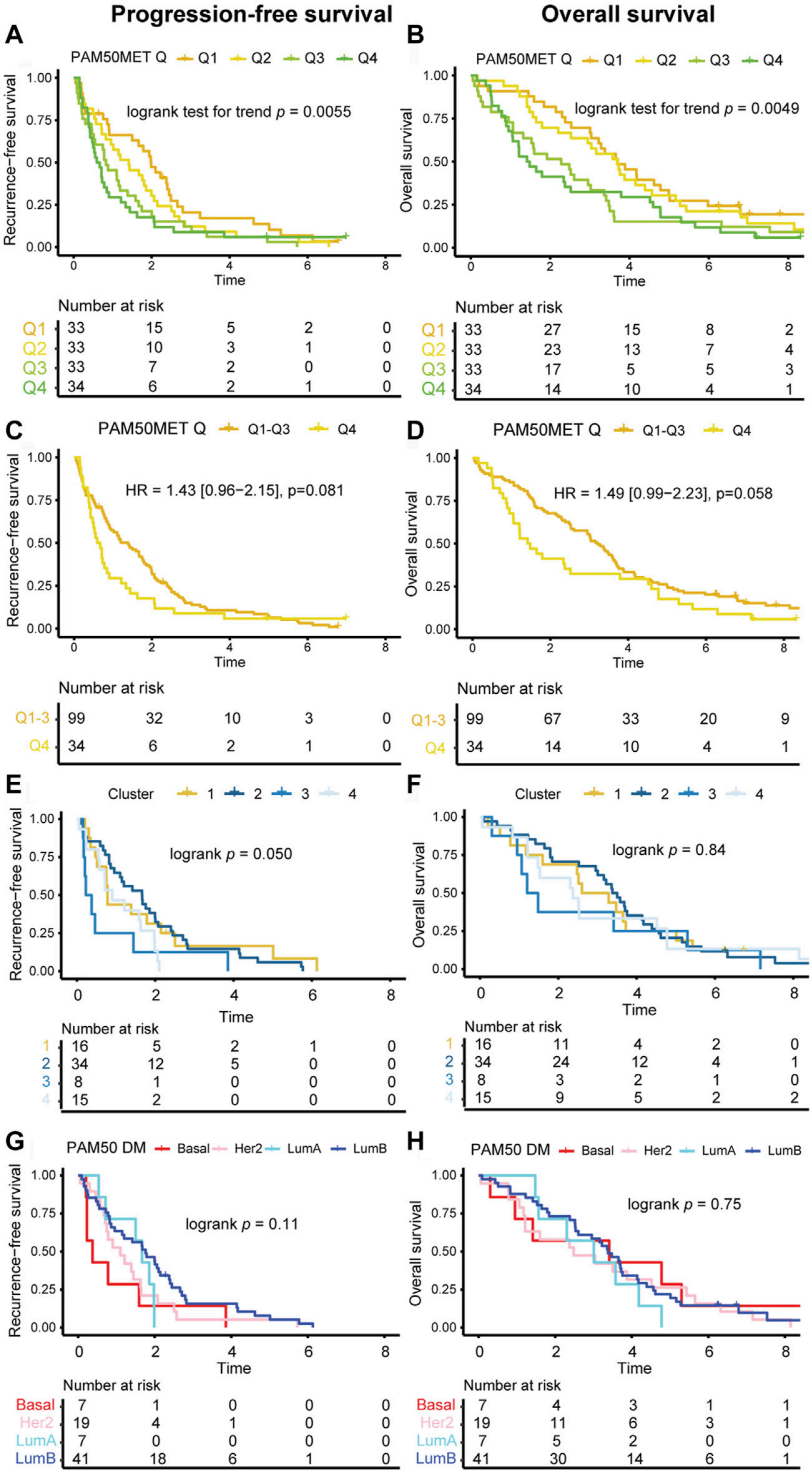
Kaplan-Meier estimates of GEX signatures. Kaplan-Meier estimates of the prognostic value in terms of PFS (left panels) and OS (right panels). Outcome in relation to **(A, B)** PAM50MET score at four levels defined by quartiles, calculated based on *n* = 72 DMs and *n* = 61 PTs, **(C, D)** PAM50MET stratified as quartile 1–3 (Q1-3) and quartile 4 (Q4), calculated based on *n* = 72 DMs and *n* = 61 PTs, **(E, F)** clusters aqcuired by applying k-means clustering on the Euclidean distance calculated based on the BC360 biological GEX signatures, **(G, H)** PAM50 subtype of the DM. Differences between groups were tested using either logrank tests (categorical nominal variables), logrank tests for trend (categorical ordinal variables), or Cox proportional hazards models (binary or continuous variables). HRs are presented with 95% CI and crude *p* values and correspond to analyses of the full follow-up. Abbreviations: CI, confidence interval; DM, distant metastasis; GEX, gene expression; HR, hazard ratio; PFS, progression-free survival; PT, primary tumor; MBC, metastatic breast cancer; OS, overall survival; Q, quartile.

The PAM50MET scores in the current study were primarily calculated based on DM GEX (*n* = 72) and based on PT GEX when DM data was not available (*n* = 61), as described by Prat et al. (2020) To evaluate the prognostic value of PAM50MET, we first assessed the scores in terms of quartiles, where a linear relationship was found between outcome and PAM50MET quartile, both for PFS (Figure 9A; logrank test for trend *p* = 0.0055) and OS (Figure 9B; logrank test for trend *p* = 0.0049). The relationship was more pronounced during the first 2 years of follow-up for both PFS (logrank test for trend *p* = 0.0008) and OS (logrank test for trend *p* = 0.0001). We also compared PAM50MET as quartile four compared to quartiles one to three, as reported in the original article (Figures 9C,D) (Prat et al., 2020). The prognostic value during the full follow-up period was limited, but during the first 2 years of follow-up, quartile four associated to an inferior prognosis both in terms of PFS (HR_2 yrs FU_ = 1.70, 95% CI = 1.09–2.66, *p* = 0.019) and OS (HR_2 yrs FU_ = 2.28, 95% CI = 1.30–4.00, *p* = 0.0040). As the PAM50MET model was trained in ER+/HER2- MBC, we also performed the prognostic analyses including only this subgroup. In this subgroup, the PAM50MET calculations were based on *n* = 56 DMs and *n* = 25 PTs. In general, the patterns were similar to what was observed in the full cohort (Supplementary Figure S8). In summary, PAM50MET showed a promising potential as a prognostic tool for MBC in this material.

### 3.10 Prognostic performance of the clusters

To address if the biological information provided by the 31 biological signatures in the BC360 panel can be used for prognostic purposes, we performed k-means clustering of the Euclidean distance based on the DM GEX of the BC360 panel. We detected four sample clusters, which were analyzed in relation to outcome. When analyzing these groups in prognosticating PFS and OS with Kaplan-Meier estimates, the clusters had a clear separation (Figures 9E,F). Visually, the ability of the clusters to identify DMs with a poor OS was higher than the DM PAM50 subtype (Figures 9E–H). In summary, the application of unsupervised machine learning revealed distinct metastatic breast cancer subtypes with prognostic implications, underscoring the crucial link between gene expression patterns and clinical outcomes.

## 4 Discussion

Using a unique material representing a timeline from primary tumor (PT) to lymph node metastasis (LNM), and distant metastasis (DM), we delineate the dynamics and prognostic relevance of gene expression (GEX) in MBC. We find that *ESR1*, *AR*, and *FOXA1* GEX expression in DM, as well as in PT, are of favorable prognostic value, and that the multigene signature representing mutant p53 is unfavorable, independently of other established prognostic factors. Notably, we find that the prognostic performance of PT GEX was at least equal to that of the DM in this cohort, indicating that the PT can provide prognostic information for MBC. As DM GEX cannot always be acquired due to practical and financial limitations, these findings are of high clinical relevance. We confirm the prognostic value of the PAM50MET model (Prat et al., 2020), which incorporates both clinical and GEX-based variables and can be performed based on GEX from the DM as well as the PT.

We validate the prognostic utility of the PAM50MET model in MBC, in terms of both PFS and OS as endpoints. Importantly, we demonstrate its applicability beyond the ER+, HER2-patient subgroup for which it was initially developed (Prat et al., 2020). In contrast to the multigene signatures evaluated in this manuscript, the PAM50MET model also integrates clinical data. While our study highlights the promising prognostic value of GEX data in MBC, it appears unlikely that GEX will entirely replace or be surrogate of clinical prognostic indicators such as the patients’ ECOG score and number of metastatic sites. This adds to the advantage of PAM50MET, as the panel illustrates how GEX can serve as a complement to clinical parameters.

In agreement with a pattern recently reported in primary breast cancer (Lundgren et al., 2023), several genes from the highly intercorrelating gene cluster associated to hormone responsiveness (*AR, PGR,* ER signaling, *ESR1,* and *FOXA1*) emerged as interesting prognostic markers for MBC, both when expressed in PT and in DM. These genes exhibited a higher expression in tumors with a favorable prognosis, independently on established prognostic markers. The strongest prognostic GEX marker in this study was *AR*, most notably in relation to PFS, and independently of other prognostic markers including DM PAM50 subtype. This suggests that *AR* GEX provides supplementary prognostic information and may have the potential to enhance the precision of MBC prognostication when integrated with the clinically established set of prognostic biomarkers. In line with prior findings (Encarnación et al., 1993; Johnston et al., 1995), we observed a downregulation of *ESR1* throughout tumor progression in endocrine-treated patients with luminal PT. However, our data reveal a decreased *ESR1* GEX in the LNM, where tumor cells remain naïve to endocrine treatment. This challenges the idea that ESR1 downregulation in breast cancer is propelled by selection pressure from endocrine treatment, suggesting that the shift from a hormone-stimulated phenotype serves additional biological purposes. One interesting idea is that growth stimulation by ER is limited in efficiency, and that prioritizing other proliferative signaling is evolutionary favorable as the tumor becomes more aggressive. Such a pattern may be more pronounced in PT and LNM of MBCs, as these could be expected to be more aggressive than the average PT and LNM.

The GEX data of this study is derived from bulk RNA. Although the tumor fractions used for GEX analyses in this study underwent macrodissection to isolate tumor tissue exclusively, this method does not exclude non-tumor cells present in the TME. This is evident in Supplementary Figure S1A, where a noticeable elevation in immune cell-related signatures in the LNM samples suggests a more prominent immune cell infiltration in this tissue. However, the relevance of non-tumor cells within the TME has gained increasing recognition, with tumor stroma playing a pivotal role in both tumor formation and progression (Xu et al., 2022). Notably, a high stromal component associates with poor outcome in several cancer types (Mesker et al., 2007; Almangush et al., 2021) including breast cancer (Roeke et al., 2017; Vangangelt et al., 2018; Vangangelt et al., 2020), underscoring the relevance of considering GEX of non-malignant, tumor-associated cells in tumor GEX analyses. Indeed, in line with previous data, the multigene signature representing T-reg cells associated to worse PFS (Walens et al., 2021). In contrast, the mast cell signature correlated positively to hormone-related genes and a favorable outcome, especially when expressed in the PT, aligning with observations in primary breast cancer (Lundgren et al., 2023). Further GEX studies employing single cell-resolution techniques retaining positional information, such as spatial transcriptomics, should be conducted to delve deeper into these concepts at a single-cell level.

The biological profile of DMs at different sites is poorly understood. We identified DM genes strongly associated to the liver and bone as metastatic sites. Interestingly, these genes align with the results from a study by Brasó‐Maristany et al., which based on the PAM50 BC360 panel, identified an 18-gene signature specific for breast cancer liver metastases (Brasó-Maristany et al., 2022) that shares a total of nine genes with the 14-gene signature from the present study. Brasó‐Maristany *et al.* also reported a 36-gene signature for bone metastases (Brasó-Maristany et al., 2022) sharing nine genes with the 21-gene cluster related to bone metastasis identified in this material. The overlap in results between studies using different methodologies strengthens the findings. While DMs at the same site expressing similar GEX profiles may not be surprising, the observation is in line with the “seed and soil” hypothesis: that distinct tumor cell clones possess varying selective affinities for metastatic sites (Paget, 1989; Langley and Fidler, 2011). However, our data cannot address the factor of causality–whether the similarities in GEX are due to natural selection, where the disseminated tumor cells must match the “soil” in order to adhere and thrive at the new site, or if the tumor cells are influenced by local factors inherent to the microenvironment once adhered to the new site. Regardless, these consistent patterns of expression at distinct metastatic sites potentially testify to a site-specific biological reliance on their functions, which could unlock new avenues for targeted treatments. In contrast, some sample clusters included DMs of various sites. This indicates that while some DM GEX profiles appear highly adapted or specialized to the metastatic site, other are tissue-agnostic and lack preferential affinity in this regard.

Our data on the relatedness of tumor triplets in terms of GEX suggest that despite similar clinical manifestations such as metastatic site, MFI, and PAM50 subtype of all three tumors, two MBCs can exhibit different patterns in terms of GEX evolution. This underlines the vast heterogeneity of MBC, and the gap of knowledge needed to be filled to be able to consider each MBC individually in outcome prediction and tailoring of systemic therapy. The international AURORA study, an initiative collecting MBC samples from paired PT and DM, circulating tumor DNA, and clinical data from 11 European countries and the United States, will provide a larger knowledgebase of temporal and spatial heterogeneity in MBC. Hopefully, this study will be a valuable contribution to the understanding MBC tumor evolution and heterogeneity, shedding light on how this can be applied to individualize treatment (Caballero et al., 2023).

After discovering that PT GEX carries relevant prognostic information regarding the outcome of MBC, we wanted to address if the PT GEX could predict future metastatic sites. Intriguingly, we identified 85 genes that were differentially expressed in PTs among patients with bone metastases at MBC diagnosis, suggesting predictive value. Therefore, we fitted a model utilizing PT GEX as predictors. The finalized model included a number of descriptors approximately tenfold higher than recommended by the Steyerberg rule-of-thumb to prevent overfitting clinical prediction models (Steyerberg, 2008). However, although the AUC estimation penalizes based on the number of descriptors, the AUC of our model drastically declined when we reduced the number of descriptors (Supplementary Table S5), indicating that a high number of genes contributed to the effective discrimination of the model in this cohort. To conclude, it is imperative to note that this is an exploratory prediction model that must be further validated in an independent dataset–preferably in a primary breast cancer cohort, as this would better simulate a clinical scenario where risk prediction of bone metastases holds practical significance. A prediction model of future metastatic site from PT GEX would be an intriguing addition to today’s prognostic factors, paving the way for individualized follow-up strategies and adjuvant therapies.

Strengths of this study include the unique cohort of 142 MBC patients previously untreated for systemic disease. The cohort has 36 PT-LNM-DM matched tumor triplets with complete GEX data, providing a unique opportunity to describe GEX dynamics through tumor progression. One weakness of the study is the cohort size. Due to limited statistical power, adjustment for false discovery rate was not performed for all analyses. Furthermore, comparison between the prognostic performances of GEX from the PT and DM is complicated by the unequal statistical power of PT samples and DM. Thus, our data should be validated in a larger MBC cohort. Another weakness is the targeted GEX analyses, as the data reported in this study only reflects a selected fraction of the full tumor transcriptomics. The BC360 panel is curated to mirror biological events of relevance for primary breast cancer, but it is not optimized for MBC. As illustrated in this study, there are profound shifts in terms of GEX from the PT to its metastases. Furthermore, with each metastatic site exhibiting such distinct GEX profiles, it is likely that the transcriptomic landscapes of each site are profoundly different, and that the GEX coverage of LNM and DM may be suboptimal.

To conclude, the study fulfilled its primary aim: to assess the prognostic value of GEX in MBC–and the secondary aim: to describe the dynamics of GEX through MBC progression. The study results suggest GEX from both PT and DM as markers of potential prognostic utility. Notably, PT GEX emerges as a potent prognostic tool, offering a valuable alternative in patients where DM samples cannot be collected or analyzed. The most profound prognostic GEX markers were *AR*, *ESR1*, and *FOXA1* for a favorable prognosis, and the multigene signature p53 for an unfavorable prognosis. We also validate the performance of the PAM50MET model in predicting PFS and OS, again supporting the use of PT GEX for predicting MBC outcome in patients without available DM biopsies. The identification of specific genes associated with liver and bone metastases, along with the tissue-agnostic GEX patterns, underscores the complexity and heterogeneity of MBC. While our findings clearly support the use of GEX expression for prognostication of MBC they also contribute to our understanding of MBC. Future research exploring MBC GEX with spatial and single-cell resolution would be highly interesting to paint a more comprehensive picture.

## Data Availability

The original contributions presented in the study are included in the article/Supplementary Material, further inquiries can be directed to the corresponding author.
