# Supplementary figures and images for: Gene expression in metastatic breast cancer—patterns in primary tumors and metastatic tissue with prognostic potential
Source: Front Mol Biosci. 2024 Feb 21;10:1343979. doi: 10.3389/fmolb.2023.1343979 (PMC10916684; doi:10.3389/fmolb.2023.1343979)

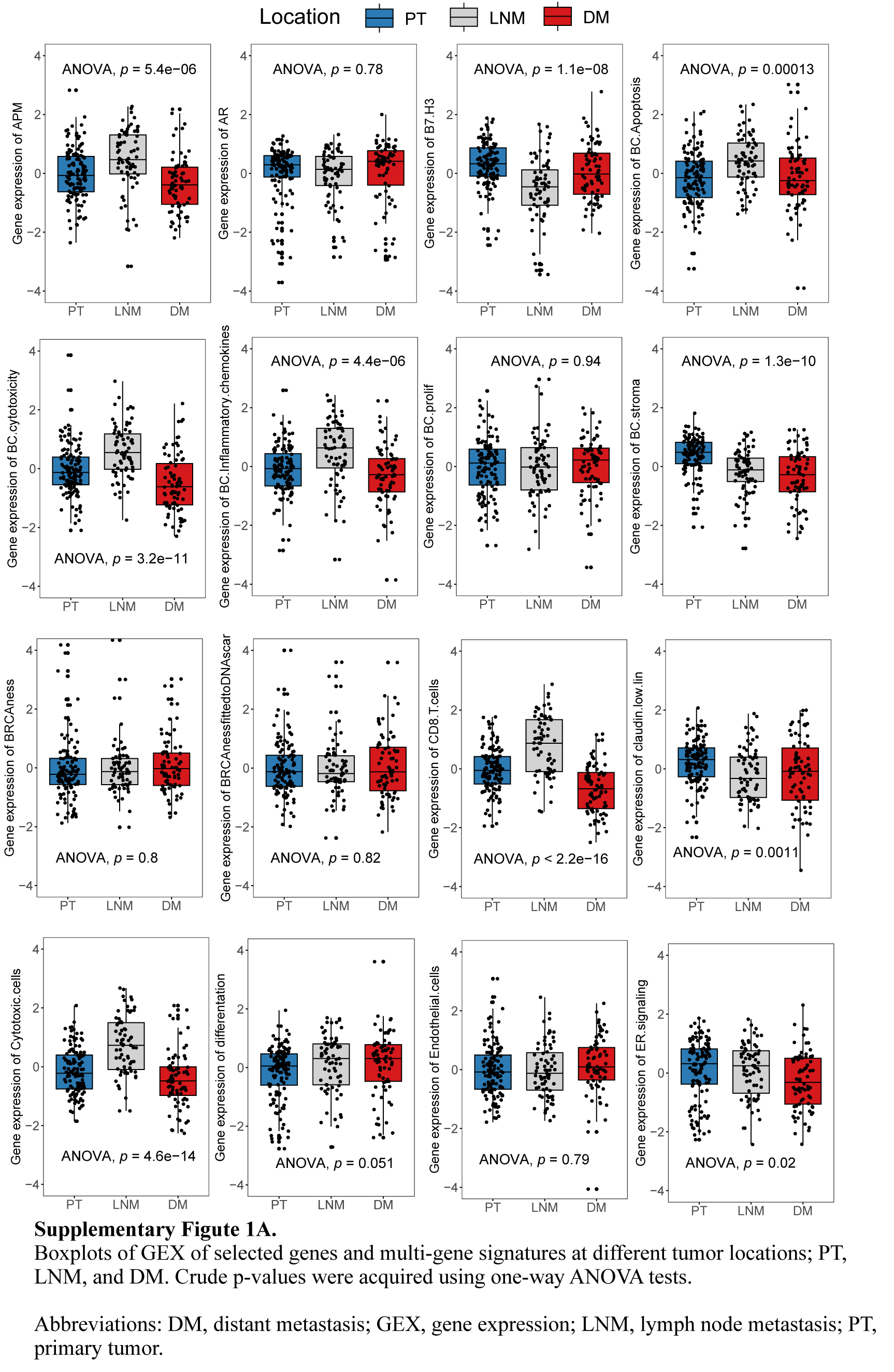

Supplement: Supplementary file 3 [file DataSheet1.zip › TIF/Supplementary Figure 1A.tif]

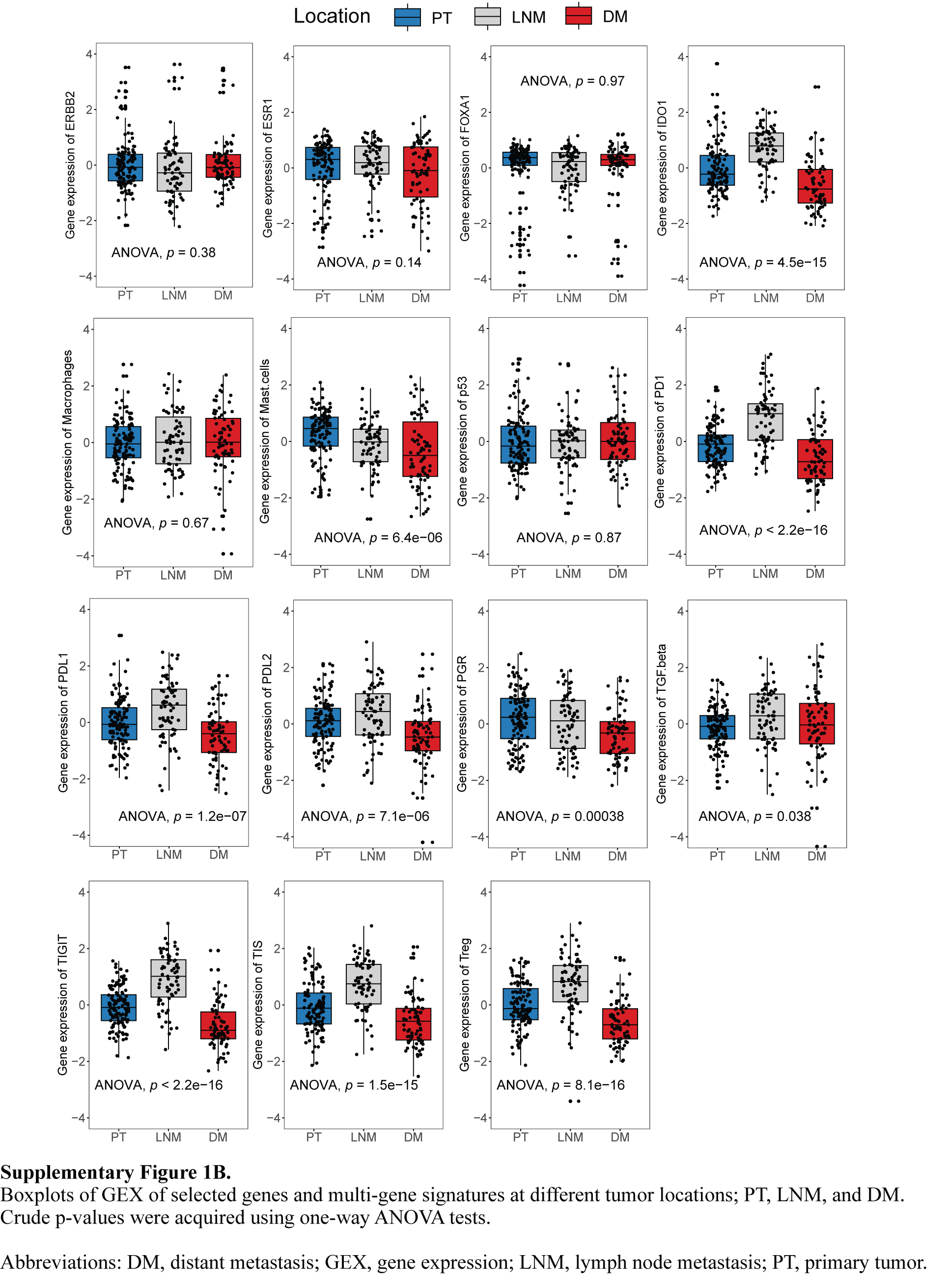

Supplement: Supplementary file 3 [file DataSheet1.zip › TIF/Supplementary Figure 1B.tif]

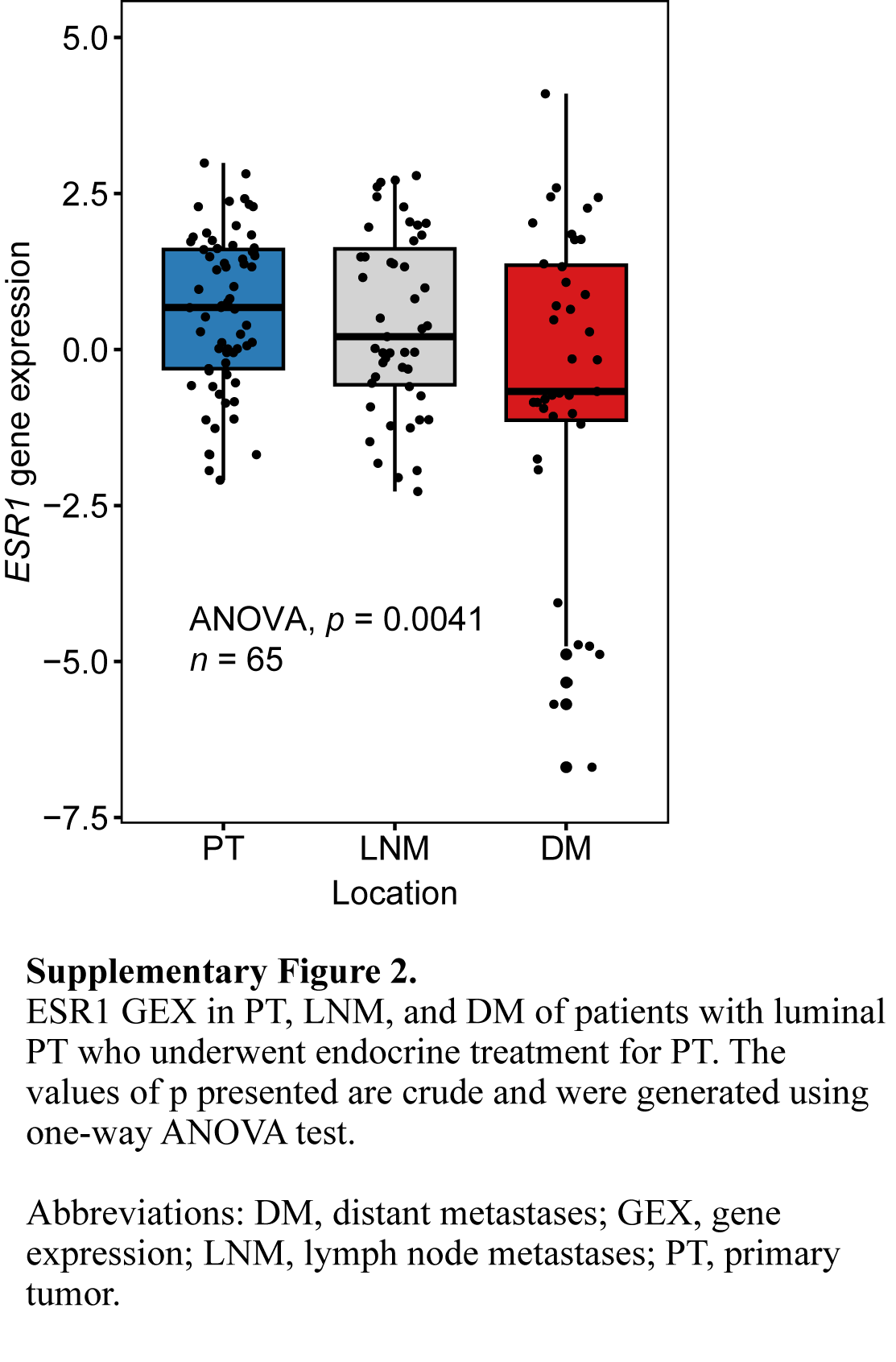

Supplement: Supplementary file 3 [file DataSheet1.zip › TIF/Supplementary Figure 2.tif]

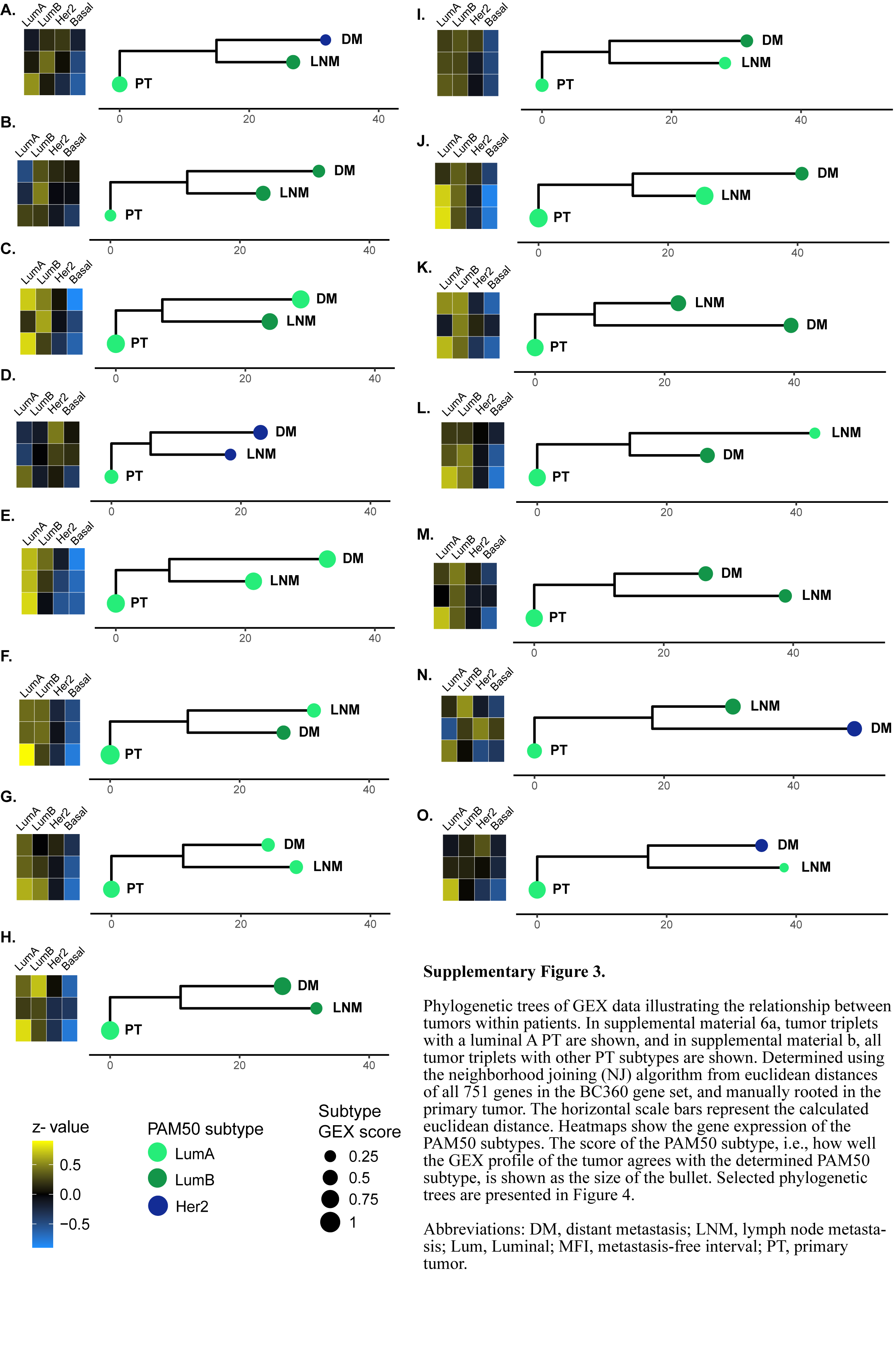

Supplement: Supplementary file 3 [file DataSheet1.zip › TIF/Supplementary Figure 3.tif]

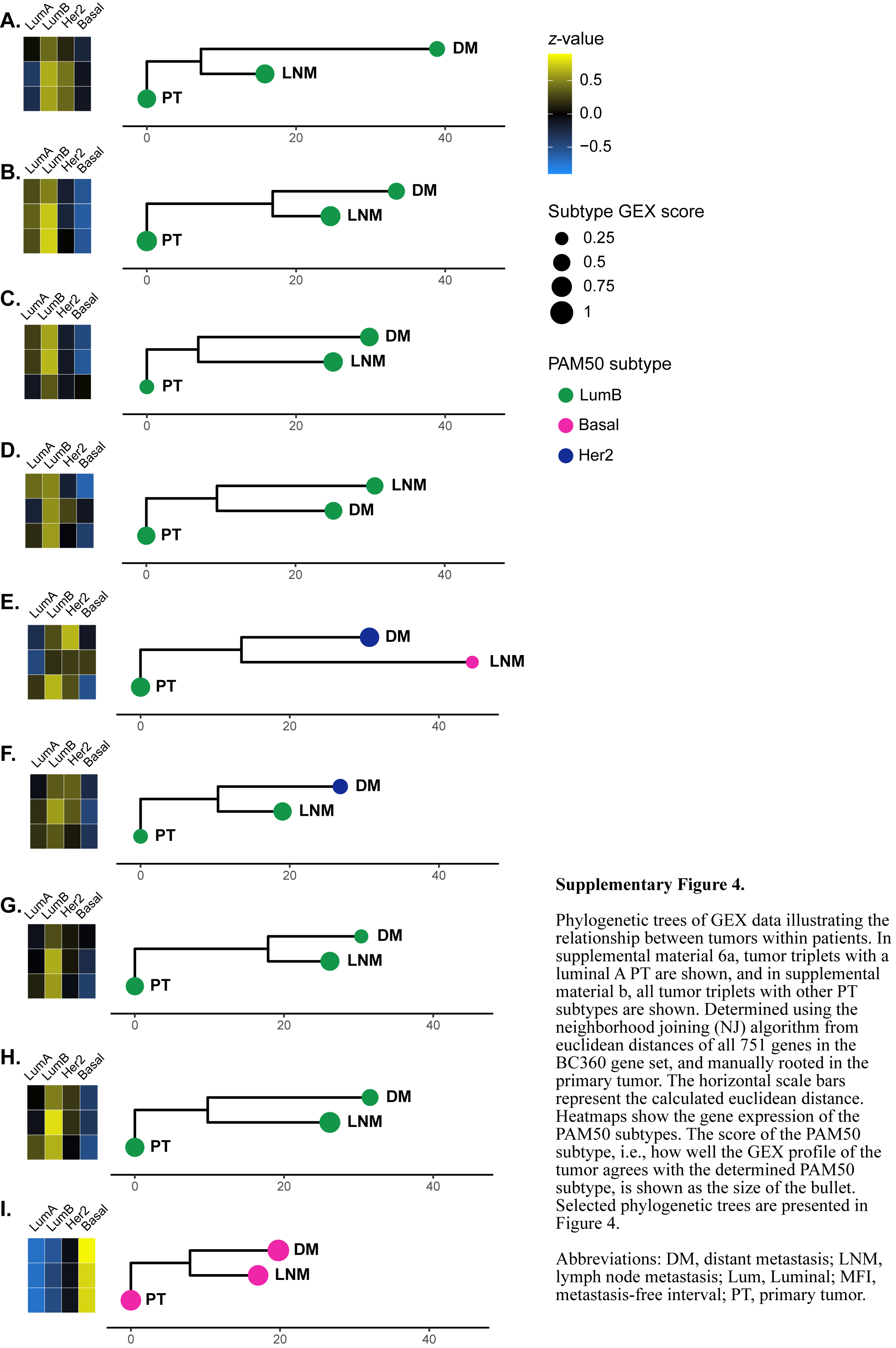

Supplement: Supplementary file 3 [file DataSheet1.zip › TIF/Supplementary Figure 4.tif]

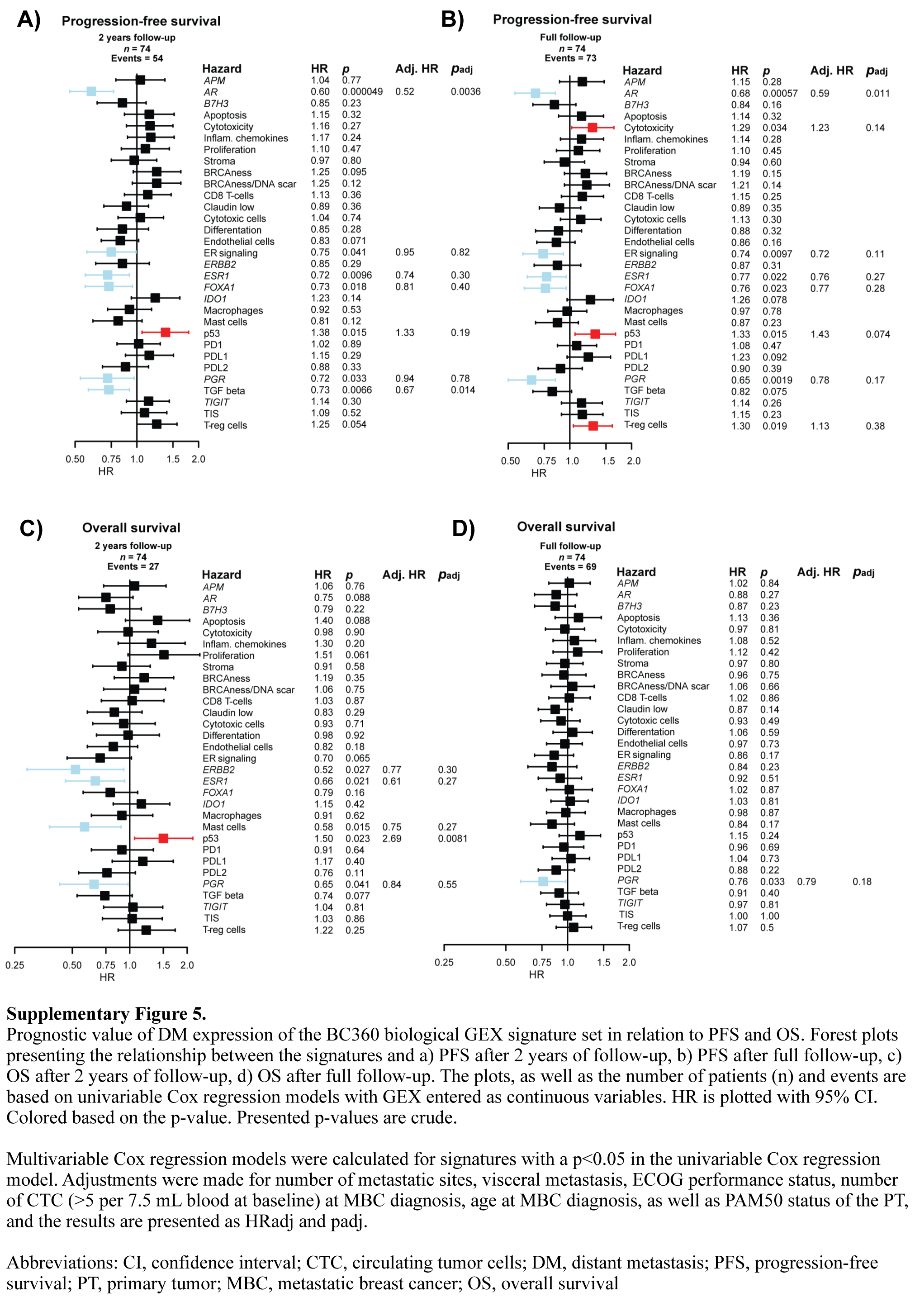

Supplement: Supplementary file 3 [file DataSheet1.zip › TIF/Supplementary Figure 5.tif]

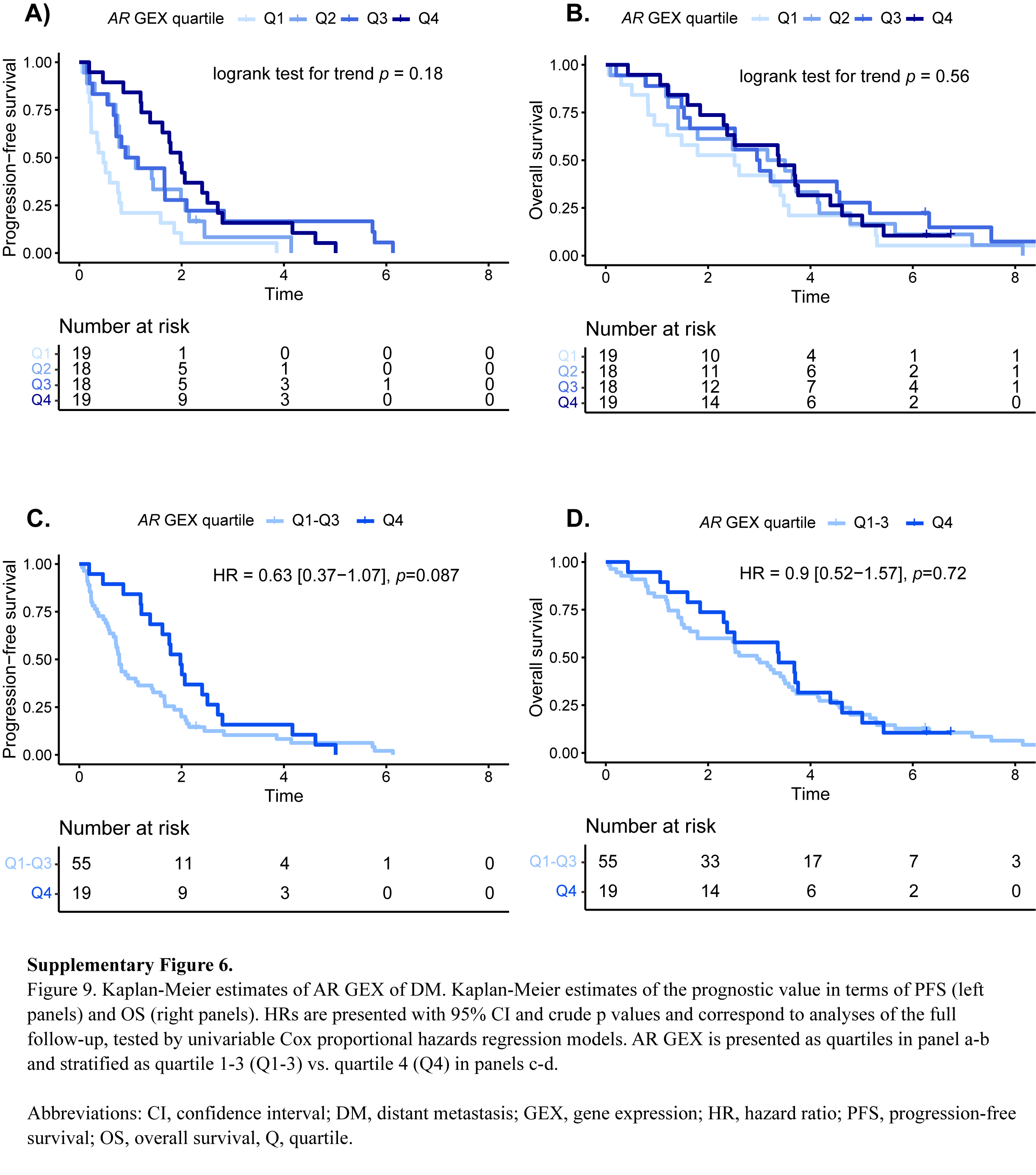

Supplement: Supplementary file 3 [file DataSheet1.zip › TIF/Supplementary Figure 6.tif]

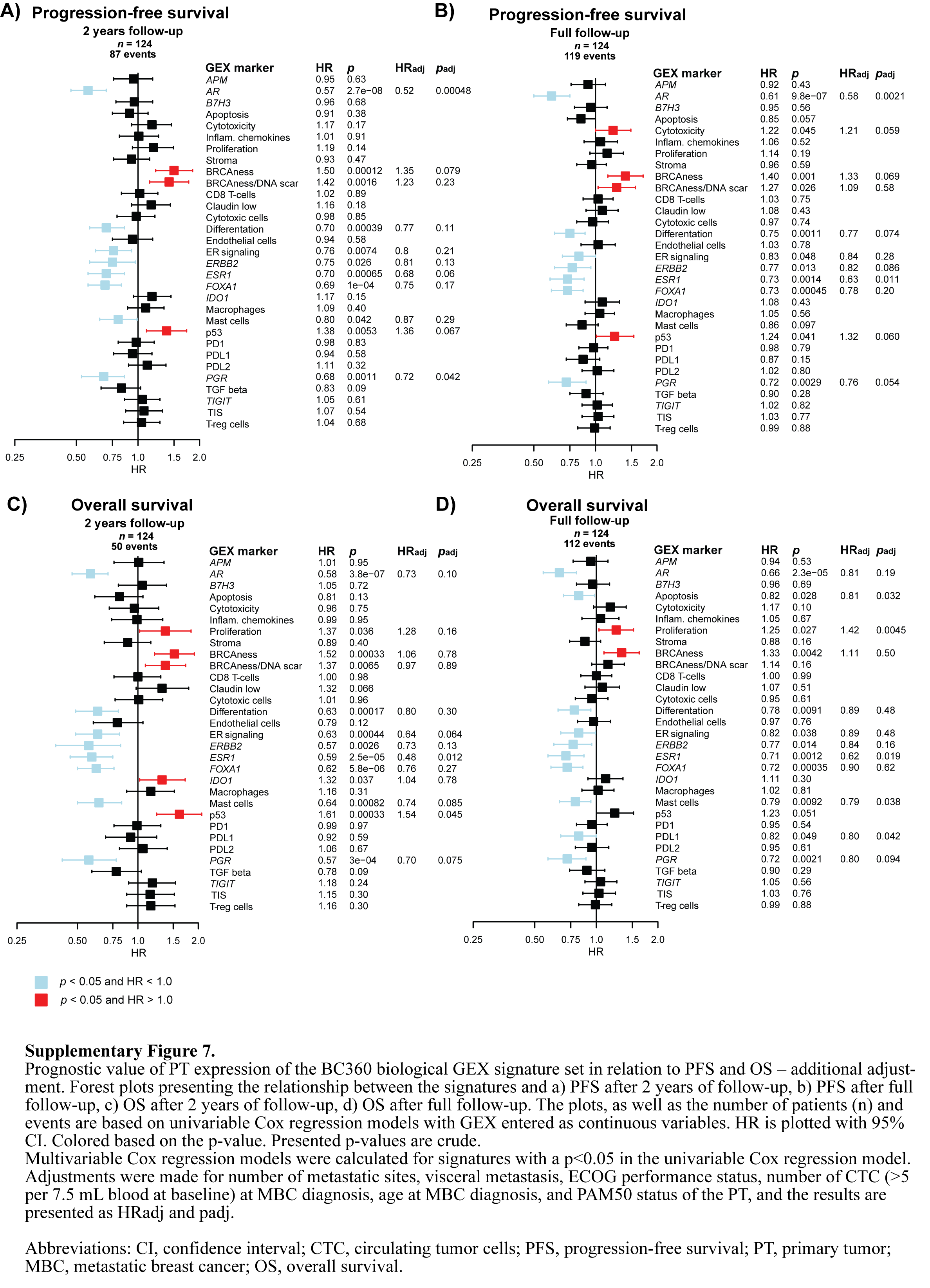

Supplement: Supplementary file 3 [file DataSheet1.zip › TIF/Supplementary Figure 7.tif]

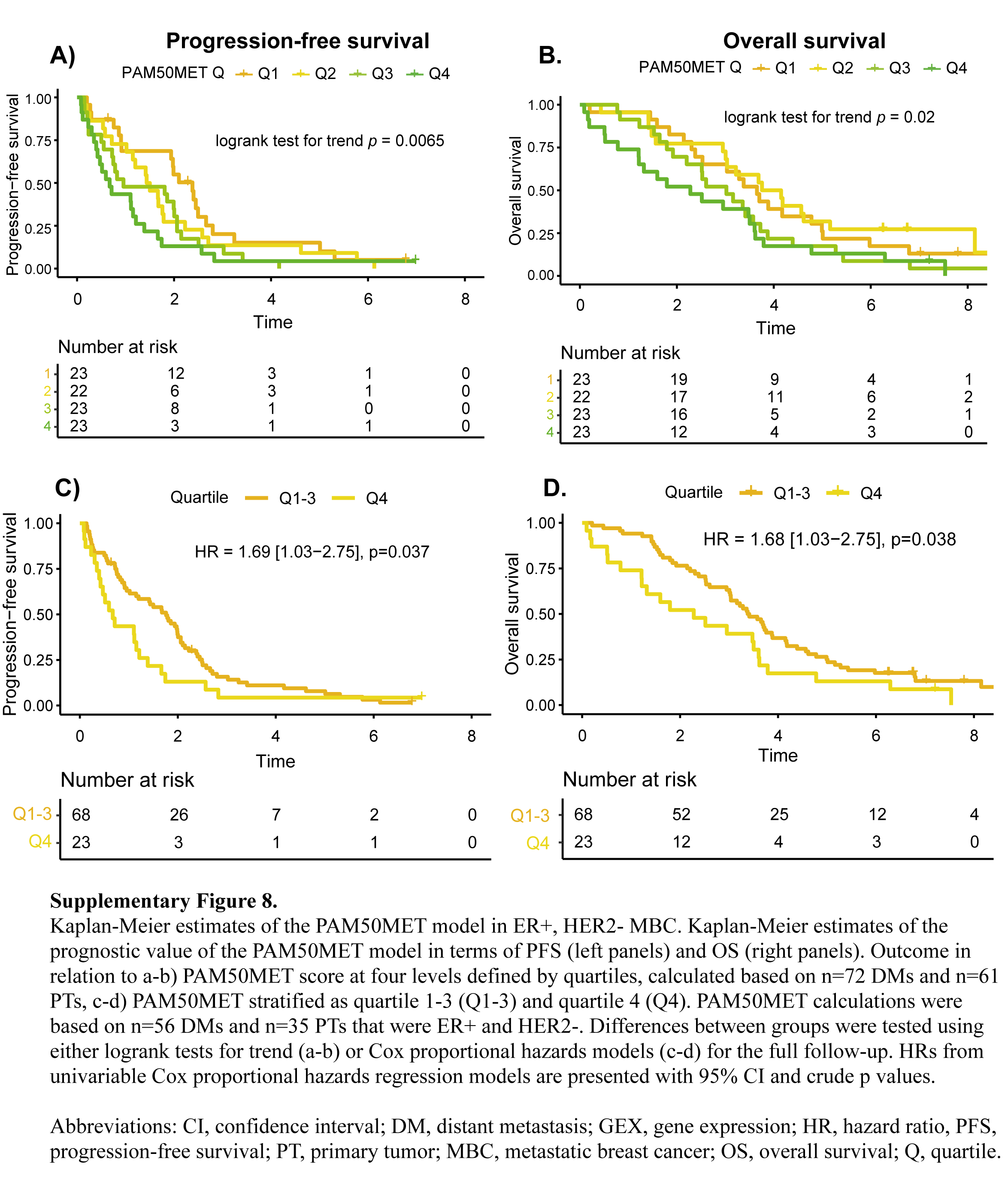

Supplement: Supplementary file 3 [file DataSheet1.zip › TIF/Supplementary Figure 8.tif]
