# Supplementary material for: Gene expression in metastatic breast cancer—patterns in primary tumors and metastatic tissue with prognostic potential
Source: Front Mol Biosci. 2024 Feb 21;10:1343979. doi: 10.3389/fmolb.2023.1343979 (PMC10916684; doi:10.3389/fmolb.2023.1343979)
Supplement: Supplementary file 4 [file Table5.docx]

### Supplementary material Table 5, .docx

Detailed information on the development of the GEX signature of PTs developing bone metastasis. Includes methodological details including full output from a stepwise logistic regression model optimizing the prediction error (Akaike information criterion; AIC) of GEX data from primary tumors, comparing GEX of primary tumors that metastasized to bone with those that did not, GO enrichment analysis of biological function of the genes of the bone metastasis model, and a ROC curve of the performance of the model. The file also contains methods and results from 100 000 iteratively randomized mock variables mimicking the distribution of the bone metastasis variable run through the modeling pipeline, performed to address the risk of model overfitting.

Adjustment of *p-*values was performed by the FDR method, creating *q*-values.

Abbreviations: AIC, Akaike information criterion; FDR, false discovery rate; GEX, gene expression.

### Results

The performance of the model in identifying the PTs that developed bone metastasis in this material was illustrated by a ROC curve with an area under curve (AUC) of 0.977 (Supplementary File S7-2). The AIC of the model was 92.5.

**Table S7-1**. Genes expressed in PTs associated to bone metastasis, full model from stepwise regression optimizing AIC. Ordered after the FDR adjusted *p*-value (*q*).

|  | **Estimate** | **Std. Error** | ***p*** | ***q*** |
| --- | --- | --- | --- | --- |
| (Intercept) | 0.653 |  |  |  |
| *BMP7* | -0.262 | 0.045 | 8.07E-08 | 1.70E-06 |
| *PRC1* | -0.309 | 0.069 | 2.55E-05 | 0.0003 |
| *PRKDC* | -0.165 | 0.038 | 3.27E-05 | 0.0003 |
| *DLL4* | 0.228 | 0.054 | 5.96E-05 | 0.0005 |
| *SLC44A4* | -0.263 | 0.064 | 9.41E-05 | 0.0007 |
| *DLGAP5* | 0.335 | 0.089 | 0.0003 | 0.002 |
| *TRIP13* | 0.236 | 0.063 | 0.0003 | 0.002 |
| *ADM* | -0.144 | 0.040 | 0.0005 | 0.002 |
| *MUC1* | -0.187 | 0.054 | 0.0008 | 0.003 |
| *SIDT1* | 0.172 | 0.055 | 0.003 | 0.010 |
| *CKS1B* | 0.197 | 0.067 | 0.004 | 0.015 |
| *ASPN* | 0.152 | 0.052 | 0.005 | 0.015 |
| *RFC4* | 0.173 | 0.061 | 0.006 | 0.017 |
| *AR* | -0.172 | 0.064 | 0.009 | 0.022 |
| *XRCC2* | -0.153 | 0.057 | 0.009 | 0.022 |
| *MIS18A* | -0.139 | 0.052 | 0.009 | 0.022 |
| *PLA2G3* | 0.108 | 0.040 | 0.009 | 0.022 |
| *ELF3* | 0.103 | 0.039 | 0.01 | 0.023 |
| *KIAA0040* | 0.122 | 0.049 | 0.01 | 0.030 |
| *TNFSF10* | 0.102 | 0.042 | 0.02 | 0.034 |
| *KIF2C* | -0.169 | 0.076 | 0.03 | 0.055 |
| *AURKB* | 0.155 | 0.070 | 0.03 | 0.055 |
| *SCUBE2* | 0.145 | 0.071 | 0.04 | 0.072 |
| *CCNE1* | 0.135 | 0.065 | 0.04 | 0.072 |
| *BMP4* | 0.094 | 0.046 | 0.04 | 0.072 |
| *DTX3* | -0.102 | 0.051 | 0.05 | 0.074 |
| *PIP* | 0.101 | 0.051 | 0.05 | 0.074 |
| *LRRC32* | -0.145 | 0.075 | 0.05 | 0.074 |
| *TTK* | 0.126 | 0.064 | 0.05 | 0.074 |
| *NFKBIZ* | -0.081 | 0.041 | 0.05 | 0.074 |
| *FAM83D* | -0.131 | 0.071 | 0.07 | 0.092 |
| *FAM214A* | 0.109 | 0.060 | 0.07 | 0.093 |
| *CCNA2* | -0.146 | 0.081 | 0.08 | 0.093 |
| *CHAD* | -0.084 | 0.051 | 0.10 | 0.124 |
| *SLC39A6* | 0.077 | 0.050 | 0.12 | 0.146 |
| *CHEK2* | -0.062 | 0.045 | 0.17 | 0.192 |
| *FAM198B* | 0.074 | 0.054 | 0.17 | 0.192 |
| *ARNT2* | 0.062 | 0.046 | 0.18 | 0.196 |
| *PDGFRB* | 0.114 | 0.090 | 0.2 | 0.215 |
| *LFNG* | 0.069 | 0.055 | 0.21 | 0.215 |
| *RB1* | -0.051 | 0.041 | 0.21 | 0.215 |


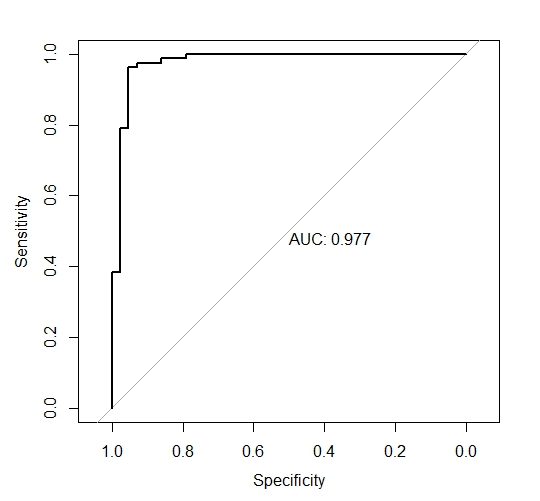


**Figure S7-1. ROC curve of the performance of the full bone metastasis model in identifying the PTs that developed bone metastasis.**

The final model from the backward stepwise logistic regression included 41 descriptors. This by far exceeds ten observations in the rarest of the two outcome categories per descriptor, which is recommended according to Steyerbergs’ rule-of-thumb to avoid overfitting (1). Thus, we fitted additional more pragmatic models, beginning at four descriptors as suggested by Steyerberg, up to 30 (Figure S7-2).

The AIC of the model with four descriptors was 129.9, as compared to the AIC of 92.5 for the full model. The fact that the larger model has both a higher AUC and a lower AIC despite AIC penalty for model complexity compared to the smaller model illustrates that the gain in performance from adding predictors to the model was substantial in this study material.


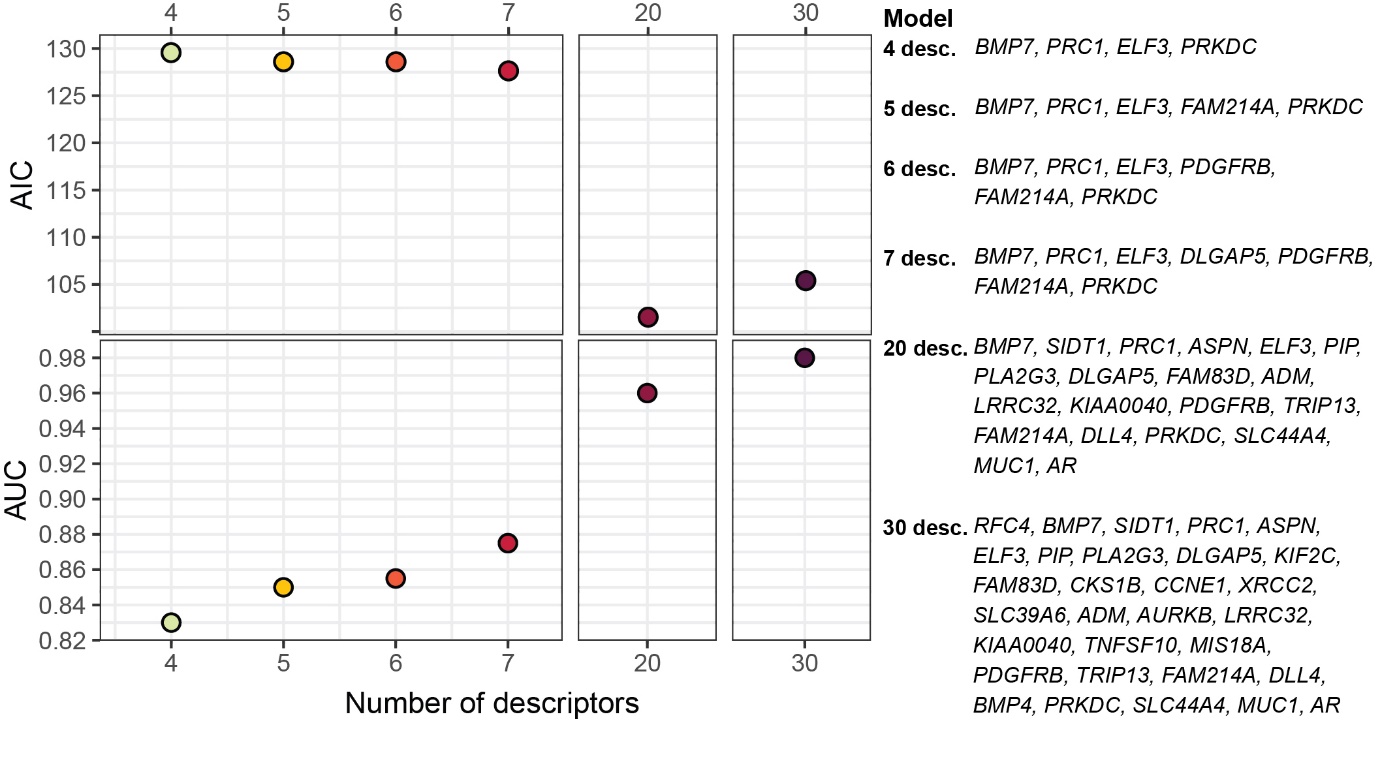


**Figure S7-2. AIC and AUC of bone metastasis models including an increasing number of descriptors**.

### AUC of mock models

To better understand the degree of overfitting of the final large model, we permuted the outcome variable 100 000 times and ran through the same pipeline for each permutation.

Of 100 000 datasets with permuted outcomes, only 3625 (3.6%) had any genes with *q*<0.05 in the differential GEX analysis, leading to continuation into the stepwise logistic regression modeling step. The distribution of AUC values of these 3625 datasets are illustrated in Figure S7-3. The mean AUC was 0.752, and the range was 0.62-1.00. The number of models leading to an AUC>0.977 was 50, corresponding to 0.05% of the 100 000 datasets generated under the null hypothesis of no association between gene expression and outcome.

In summary, these data suggest that the pipeline of differential gene expression analysis followed by stepwise logistic regression modeling leads to a final prediction model with a discrimination (AUC) which is reached in only 0.05% of the 100 000 final models fitted under the null hypothesis of no correlation between GEX data and the outcome. The risk of overfit is, however, large in a small sample like this with so many candidate predictors.


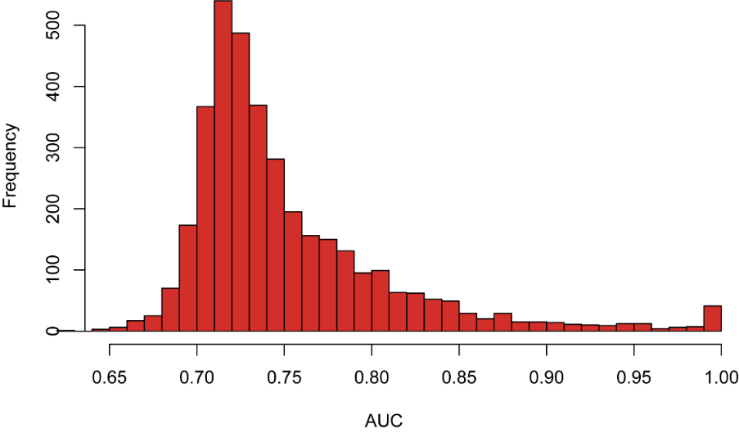


**Figure S7-3. Distribution of AUC of models with mock variables**.


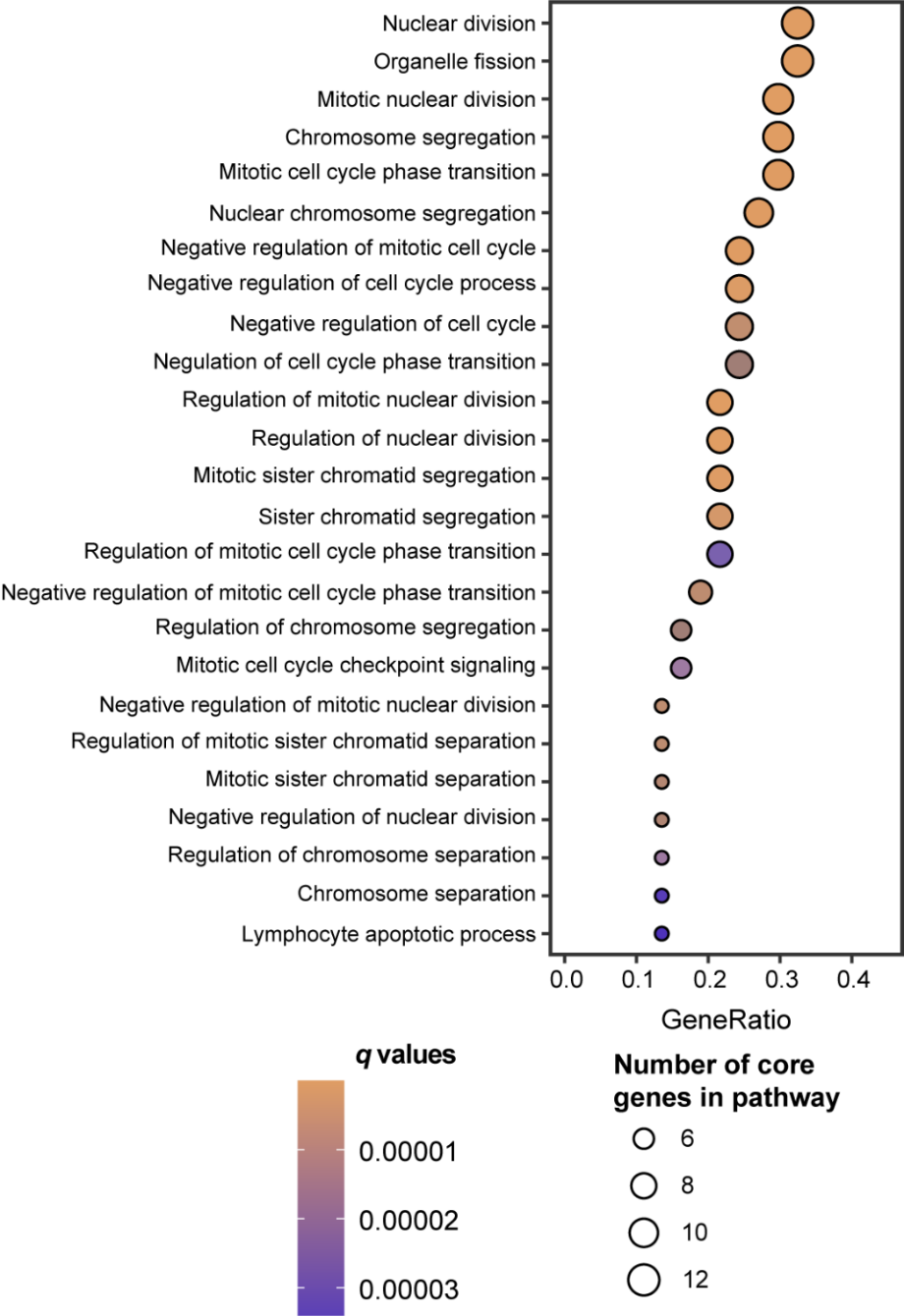


**Figure S7-4. Gene Ontology (GO) enrichment analysis of biological function of the genes of the bone metastasis model**, trained to identify primary tumors (PT) with potential to metastasize to bone. Showing the top 25 biological functions based on GeneRatio, which is the ratio between core enrichment genes and the total number of genes in the pathway and illustrates to which level the gene cluster is altered in the pathway. The number of core enrichment genes is indicated by the dot size. The *q* values represent *p* values adjusted for FDR.

1. Steyerberg EW, SpringerLink. Clinical prediction models : a practical approach to development, validation, and updating. Cham: Springer International Publishing : Imprint: Springer; 2008.
