# Supplementary material for: Gene expression in metastatic breast cancer—patterns in primary tumors and metastatic tissue with prognostic potential
Source: Front Mol Biosci. 2024 Feb 21;10:1343979. doi: 10.3389/fmolb.2023.1343979 (PMC10916684; doi:10.3389/fmolb.2023.1343979)
Supplement: Supplementary file 6 [file Table4.docx]

### Supplementary material Table 4, .docx

The variables and coefficients used in the calculation of the PAM50MET score. Also described in the original publication (8).

| Coefficient | Variable | Type |
| --- | --- | --- |
| 0.178 | Performance_status_0 | Binary |
| -0.160 | Luminal A | Continuous |
| -0.066 | *SLC39A6* | Continuous |
| -0.036 | *MAPT* | Continuous |
| -0.012 | *PGR* | Continuous |
| -0.008 | *NAT1* | Continuous |
| -0.003 | *ESR1* | Continuous |
| -0.003 | Age at DM diagnosis | Continuous |
| -0.002 | *TMEM45B* | Continuous |
| 0.265 | >3 metastatic sites | Binary |
| 0.147 | GEX data from PT instead of DM | Binary |
| 0.678 | Her2-enriched PAM50 subtype | Binary |
| 0.444 | Basal | Continuous |
| 0.131 | *CCNB1* | Continuous |
| 0.107 | *PHGDH* | Continuous |
| 0.050 | *FGFR4* | Continuous |
| 0.038 | *GRB7* | Continuous |
| 0.020 | *FOXA1* | Continuous |
| 0.018 | *NUF2* | Continuous |
| 0.011 | *GPR160* | Continuous |
| 0.003 | *UBE2T* | Continuous |
| Description from the original article*: “The PAM50MET variables associated with a better PFS (i.e., low PAM50MET scores) were ECOG PS of 0 (versus 1), older age, PAM50 luminal A subtype (vs. not), and high expression of the following genes: solute carrier family 39 member 6 (SCL39A6), also known as LIV-1, microtubule associated protein tau (MAPT), progesterone receptor (PGR), N-acetyltransferase 1 (NAT1), estrogen receptor 1 (ESR1), and transmembrane protein 45B (TMEM45B). The PAM50MET variables associated with a worse PFS (i.e., high PAM50MET scores) were ≥3 metastatic sites (vs. <3 metastatic sites), primary tumor (vs. metastatic tumor), PAM50 HER2-enriched subtype, PAM50 basal-like signature score, and high expression of the following genes: cyclin B1 (CCNB1), phosphoglycerate dehydrogenase (PHGDH), fibroblast growth factor receptor 4 (FGFR4), growth factor receptor bound protein 7 (GRB7), forkhead box A1 (FOXA1), NUF2 component of NDC80 kinetochore complex (NUF2), G protein–coupled receptor 160 (GPR160), and ubiquitin conjugating enzyme E2 T (UBE2T).”* | | |

**
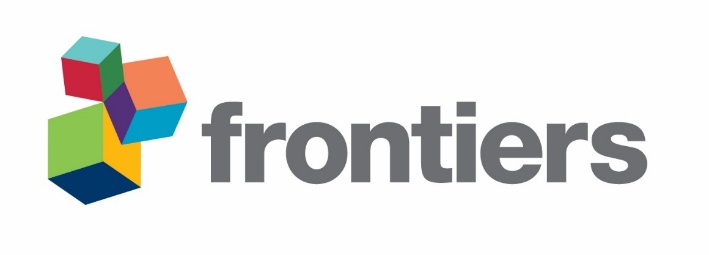
**
